# Reduced levels of NGF shift astrocytes toward a neurotoxic phenotype

**DOI:** 10.3389/fcell.2023.1165125

**Published:** 2023-04-18

**Authors:** Alexia Tiberi, Nicola Maria Carucci, Giovanna Testa, Caterina Rizzi, Paola Pacifico, Giulia Borgonovo, Ivan Arisi, Mara D’Onofrio, Rossella Brandi, Wen-Biao Gan, Simona Capsoni, Antonino Cattaneo

**Affiliations:** ^1^ BIO@SNS, Scuola Normale Superiore, Pisa, Italy; ^2^ Skirball Institute of Biomolecular Medicine, Langone Medical Center, New York University, New York, NY, United States; ^3^ European Brain Research Institute - Fondazione Rita Levi-Montalcini, Rome, Italy; ^4^ Shenzhen Bay Laboratory, Shenzhen, China; ^5^ Institute of Physiology, Department of Neuroscience and Rehabilitation, University of Ferrara, Ferrara, Italy

**Keywords:** astrocytes, NGF, calcium, co-cultures, reactive astrocytes, neurotrophins, neurotoxicity

## Abstract

Nerve growth factor (NGF) is critical for neuronal physiology during development and adulthood. Despite the well-recognized effect of NGF on neurons, less is known about whether NGF can actually affect other cell types in the central nervous system (CNS). In this work, we show that astrocytes are susceptible to changes in ambient levels of NGF. First, we observe that interfering with NGF signaling *in vivo via* the constitutive expression of an antiNGF antibody induces astrocytic atrophy. A similar asthenic phenotype is encountered in an uncleavable proNGF transgenic mouse model (TgproNGF#72), effectively increasing the brain proNGF levels. To examine whether this effect on astrocytes is cell-autonomous, we cultured wild-type primary astrocytes in the presence of antiNGF antibodies, uncovering that a short incubation period is sufficient to potently and rapidly trigger calcium oscillations. Acute induction of calcium oscillations by antiNGF antibodies is followed by progressive morphological changes similar to those observed in antiNGF AD11 mice. Conversely, incubation with mature NGF has no effect on either calcium activity nor on astrocytic morphology. At longer timescales, transcriptomic analysis revealed that NGF-deprived astrocytes acquire a proinflammatory profile. In particular, antiNGF-treated astrocytes show upregulation of neurotoxic transcripts and downregulation of neuroprotective mRNAs. Consistent with that data, culturing wild-type neurons in the presence of NGF-deprived astrocytes leads to neuronal cell death. Finally, we report that in both awake and anesthetized mice*,* astrocytes in layer I of the motor cortex respond with an increase in calcium activity to acute NGF inhibition using either NGF-neutralizing antibodies or a TrkA-Fc NGF scavenger. Moreover, *in vivo* calcium imaging in the cortex of the 5xFAD neurodegeneration mouse model shows an increased level of spontaneous calcium activity in astrocytes, which is significantly reduced after acute administration of NGF. In conclusion, we unveil a novel neurotoxic mechanism driven by astrocytes, triggered by their sensing and reacting to changes in the levels of ambient NGF.

## 1 Introduction

Astrocytes have a unique morphology that intimately reflects their function in the central nervous system (CNS). With their complex arborization and anatomical specialization, they retain an ideal position to appreciate changes in their microenvironment (Zhou et al., 2019). Some astrocyte processes closely ensheath synapses, allowing for direct modulation of the contents of the synaptic cleft, whereas others contact blood vessels *via* a specialized process called endfeet, a direct morphological correlate of their primary role in neurovascular coupling (Iadecola and Nedergaard, 2007; Santello et al., 2019). While their structural, metabolic, and homeostatic capacities have been reiterated and redefined over the years (Allaman et al., 2011; Lee et al., 2022), astrocytes have now gained an added layer of functional complexity: they are capable of directly affecting neuronal activity and, thus, the whole brain circuitry contributing to behavior (Santello et al., 2019; Kofuji and Araque, 2021). Similarly, new evidence has come forth, highlighting how neurotrophins, molecules once thought to be a prerogative of neurons, can actually be important for the physiology of astroglia as well (Bergami et al., 2008; Vignoli et al., 2016). However, little is known about the effect of nerve growth factor (NGF) (Levi-Montalcini, 1987) on astrocytes.

Mature NGF (mNGF) is synthesized as a precursor protein (proNGF) and signals *via* a dual-receptor system, comprising tropomyosin receptor kinase A (TrkA) and p75^NTR^ (Chao, 2003). While mNGF promotes neuronal survival by preferentially binding to TrkA, proNGF shows a higher affinity to p75^NTR^ and can induce apoptotic signaling in particular cellular contexts (Beattie et al., 2002; Marchetti et al., 2019). Under homeostatic conditions, proNGF and mNGF act synergistically through TrkA/p75^NTR^; however, imbalances in the relative abundances of TrkA and p75^NTR^ (or of their ligands mNGF/proNGF) have been described in multiple CNS disorders (Fahnestock et al., 2001; Tiveron et al., 2013), suggesting how tipping the scale in favor of proNGF/p75^NTR^ signaling can lead to neurodegeneration and cell death. One example of an NGF/proNGF imbalance model is the AD11 mouse transgenic line, which constitutively expresses an antibody selectively neutralizing mNGF with respect to proNGF. This leads to a progressive and comprehensive Alzheimer-like neurodegeneration, encompassing cholinergic deficits, Aβ neuropathology, hyperphosphorylation of tau, neuroinflammation, synaptic plasticity alterations, and behavioral deficits (Capsoni et al., 2000; Ruberti et al., 2000). Similarly, in a transgenic model expressing cleavage-resistant proNGF (TgproNGF#72), learning and memory impairments, cholinergic deficit, and elevated Aβ-peptide immunoreactivity can be detected (Tiveron et al., 2013). Thus, interfering with the NGF/proNGF balance can lead to neurodegeneration, suggesting a vicious cycle linking NGF dysmetabolism and Alzheimer’s disease (Capsoni and Cattaneo, 2006; Florencia Iulita and Claudio Cuello, 2015; Cuello et al., 2019).

The cellular basis for this NGF/proNGF imbalance-driven neurodegeneration goes beyond the well-established neuronal NGF–target population in the brain, namely, basal forebrain cholinergic neurons. For instance, microglia have emerged as a new target cell of NGF in the brain (Rizzi et al., 2018; Tiberi et al., 2022). These glial cells are steered toward a neuroprotective phenotype by the application of the mature neurotrophin, in particular, during amyloid-driven neurodegenerative conditions. On the other hand, limited information is present regarding the interplay between the NGF signaling system and brain astrocytes, in physiologic and pathological conditions (Cragnolini et al., 2009; Cragnolini et al., 2012; Cragnolini et al., 2018). In this work, we aim to understand the effects and consequences of the modulation of NGF levels on astrocytes and present data suggesting that astrocytes are sensors of ambient NGF levels and actively respond to reduced NGF levels by orchestrating a neurotoxic response.

## 2 Materials and methods

### 2.1 Mice

C57BL/6 × 129 (used for primary cultures), C57BL/6J, p75^NTR−/−^ (JAX strain #:002213), TrkA^+/−^ (also known as NTRK1^+/−^) (JAX strain #:002480), 5xFAD (034840-JAX), and 3xTg (MMRRC strain #034830-JAX) mice were purchased from The Jackson Laboratory (Bar Harbor, ME). AD11 mice (Ruberti et al., 2000) and TgproNGF#72 (Tiveron et al., 2013) were obtained from an in-house colony. Mice were maintained at 22°C ± 2°C with a 12-h light–dark cycle. Food and water were available *ad libitum*.

Experiments on C57BL/6 × 129, p75^NTR−/−^, NTRK1, AD11, TgproNGF#72, and 3xTG mice were performed according to the national and international laws for laboratory animal welfare and experimentation (EU directive n. 2010/63/EU and Italian DL n. 26 04/03/2014) and approved by the Italian Ministry of Health. Two-photon imaging experiments on C57BL/6J and 5xFAD mice were conducted at the New York University Medical Center (NYUMC) and approved by the Institutional Animal Care and Use Committee (IACUC) at the NYUMC*.*

For the NTRK1, AD11, TgproNGF#72, 5xFAD, and 3xTG lines, as controls, we used wild-type littermates derived from heterozygous transgenic crossing. For p75^NTR−/−^ mice, as controls, we used C57BL/6 J mice from a separate colony. Animals of both sexes were used for all experiments.

### 2.2 Intranasal delivery of NGF

Mouse NGF (or vehicle) was administered at a dose of 0.48 μg/kg intranasally to 1.5-month-old 3xTG mice every 2 days for a 15-day period. Briefly, the peptide was diluted in 1 M phosphate-buffered saline (PBS, 137 mM NaCl, 2.7 mM KCl, 10 mM Na_2_HPO_,4_ 1.8 mM K_2_HPO_4_ pH 7.4) and administered intranasally to the mice, by providing 3 µl of total volume per puff, alternating nostril, and allowing enough time between each administration (around 2 min), as described by Capsoni et al. (2002).

### 2.3 Immunofluorescence on brain slices for detection of astrocyte morphology

The mice received terminal anesthesia (chloral hydrate, 10 mg/kg) and underwent a perfusion procedure. First, they transcardially received ice-cold phosphate-buffered saline (PBS) to wash out blood from the system. Then, ice-cold 4% paraformaldehyde (PFA) was added to 0.1 M pH 7.4 phosphate-buffered saline. After dissection, the brain was post-fixed for 48 h and cryoprotected with 30% sucrose (in PBS) before cutting 50-µm coronal sections using a cryostat microtome (Leica Microsystems, Wetzlar, Germany). The sections were stained overnight at 4°C with either anti-glial fibrillary acidic protein (GFAP) (DakoCytomation, Glostrup, Denmark, Z0334, 1:500) or goat anti-GFAP (Santa Cruz Biotechnology, California, United States, sc-6170, 1:300). The appropriate secondary antibodies (Thermo Fisher Scientific, MA, United States; A-21428 diluted 1:500) were used for 2 h at RT. Images from WT, AD11, TgproNGF#72, p75^NTR−/−^, and NTRK1^+/−^ mice were acquired using a confocal laser scanning microscope (TCS SP2; Leica Microsystems, Wetzlar, Germany), equipped with an oil objective (HCX PL APO ×63.0 OIL (NA = 1.40)). Then, 50-µm stacks in the stratum moleculare of the hippocampus were acquired for analysis, keeping the pinhole at 1 AU. Image processing was performed using the filament function *via* the Imaris Bitplane software. Reconstruction was performed using 8-μm spheres for the soma and 0.400-μm spheres for reconstructing the branches. Parameters such as Sholl intersections, filament lengths, number of branching points, and number of terminal points were measured.

### 2.4 NGF ELISA

NGF levels in AD11 brain samples and primary astrocyte supernatant were measured using an ELISA kit that employs an antibody specific for human beta NGF that detects both mature and immature NGF (ELISA NGF Emax ^®^ ImmunoAssay System, Promega). Brain tissue (0.1–0.5 g) or whole primary astrocyte supernatant was extracted in 1–2 ml of ice-cold buffer (125 NaCl, 50 mM Tris, 1 mM EDTA, 1 mM EGTA, 0.5% Nonidet P-40, pH 7.4, and protease inhibitors (cOmplete™ Protease Inhibitor Cocktail, MERK)). After sonication, the samples were centrifuged at 16,000 rpm at 4°C, and the supernatant was collected and centrifuged again at the same speed and temperature, which was then processed according to the manufacturer’s instructions.

### 2.5 Purification of antibodies

The antiNGF rat IgG2a mAbaD11 (Cattaneo et al., 1988; Covaceuszach et al., 2012) was purified from the serum-free supernatant of the corresponding hybridoma cell line, after precipitating with 29% ammonium sulfate, followed by affinity chromatography using a Protein G Sepharose column (Pharmacia) and elution with low-pH buffer (10 mM HCl). mAb aD11 IgG2a fractions were pooled and dialyzed against 10 mM of sodium phosphate pH 7.0 and 20 mM of EDTA. Single-chain Fv fragment scFv A13 (Meli et al., 2009), recognizing A beta oligomers, was purified from E. coli cells (strain BL21 (DE3)pLysS (Novagen)) after oxidative refolding, as described (Meli et al., 2014). scFv A13 was provided by Giovanni Meli/EBRI.

### 2.6 Primary astrocyte cultures

Postnatal day 3–4 C57BL/6 × 129 mice were decapitated, the brain was quickly excised, and the hippocampus was dissected. The cells were maintained in Dulbecco’s modified Eagle’s medium (DMEM/F12) (Thermo Fisher Scientific, MA, United States #21331-020) containing 1% penicillin/streptomycin (Euroclone, MI, Italy #ECB3001D), 1% GlutaMAX (Thermo Fisher Scientific, MA, United States; #35050-038), and 10% fetal bovine serum (FBS) (Euroclone, MI, Italy #ECS0180l) in 5% CO_2_ pH 7.4 at 37°C. Mild shaking was performed to eliminate microglia from the mixed primary glial cultures. At DIV 14, astrocytes were detached by mild trypsinization for 10 min with 0.01% of trypsin (Thermo Fisher Scientific, MA, United States), and, after proper resuspension in the aforementioned culture medium, they were plated on the appropriate support, depending on the type of experiment, and allowed to rest for 24 h before use. The purity of our astrocyte culture was assessed to be 98% on the basis of cell-type markers (in line with Schildge et al. (2013)).

#### 2.6.1 Immunocytochemistry analysis of astrocytes

Primary astrocytes were seeded on coverslips in 24-well plates coated with poly-D-lysine at a density of 1 × 10^5^ cells/well and were treated for 48 h with either NGF (100 ng/ml), antiV5 (800 ng/ml), or increasing doses of αD11. After fixation with 2% PFA (for 10 min) and blocking (1 h at RT), the samples were stained with primary antibodies overnight at 4°C: rabbit anti-GFAP (DakoCytomation, Glostrup, Denmark, Z0334, 1: 500) or goat anti-GFAP (Santa Cruz Biotechnology, California, United States, sc-6170, 1:300). The appropriate secondary antibodies were then incubated for 1 h at RT (Thermo Fisher Scientific, MA, United States; A-21428; A-21201, 1:500). Fluoroshield (Sigma Aldrich, MO, United States #F6057) containing DAPI was used to mount the coverslip, and a Leica SP2 confocal microscope (Leica Microsystems, Wetzlar, Germany) equipped with an HCX PL APO 63.0X OIL (NA = 1.40) objective was used to acquire images.

#### 2.6.2 Calcium imaging in astrocytes *in vitro*

Oregon Green™ 488 BAPTA-2 (O6809, Thermo Fisher) (1 µM) was used as the calcium indicator to detect calcium transients. Astrocytes plated in an imaging well were treated with the indicator for 20 min and then washed carefully as specified in the datasheet.

Due to the non-ratiometric inherence of the Oregon Green™ 488 BAPTA-2, we report the results as the percentage for each frame of active cells (around 60 active cells per recording session were found active). A cell was considered active if its activity increased by 5% compared to the average brightness of the same cell in the previous 15 and subsequent 15 frames.

Imaging was performed with an acquisition frequency of 0.2 Hz (one frame every 5 s). After 250 frames of baseline recording (corresponding to 20 min), we added antiV5 antibody (800 ng/ml), αD11 (800 ng/ml), or murine NGF (100 ng/ml) and performed imaging up to 1,000 frames (1 h). Thapsigargin was used at a concentration of 1 µM, and it was added 30 min before imaging. Drugs were diluted in 20 µl of KREBS salt solution and gently added to the Petri dish with minimal tapping, ensuring such application is performed far from the focused registration area, for a total volume of the recording chamber of 3 ml. Due to the fact that astrocytes express mechanosensors that can modulate their calcium activity (Shibasaki et al., 2013), recordings were conducted under still medium conditions.

### 2.7 Transcriptomic analysis

#### 2.7.1 Sample preparation and processing

Primary astrocyte culture cells were treated with αD11 (800 ng/ml), antiV5 (800 ng/ml), or vehicle (naive) for 24 h. Total RNA was extracted at 24 h from αD11-treated (test samples, *n* = 4), antiV5-treated (controls type 1, *n* = 2), and untreated naive cells (controls type 2, *n* = 2). TRIzol- (Invitrogen) and DNAse-treated Qiagen columns were used to extract RNA. The quality and integrity of samples were examined using the Agilent BioAnalyzer 2100 system (Agilent RNA 6000 Nano Kit): samples with an RNA integrity number (RIN) index lower than 8.0 were discarded, according to the standard one-color Agilent microarray gene expression protocol.

#### 2.7.2 Data processing and bioinformatics analysis

The Agilent Scanner G2564C was used for post-hybridization image acquisition. Agilent Feature Extraction version 10.7 software using the Agilent one-color gene expression extraction protocol parameters (ver GE1_107_Sep09) was used to extract data from the 20-bit TIFF images. Data quality filtering was performed using R-Bioconductor, discarding features with the flag gIsWellAboveBG = 0 in all samples. We normalized the data to the 75th percentile in the Log2 scale. A combination of fold change and moderated *t*-test thresholds (with FDR *p*-value correction) by the R-Bioconductor tool Limma (FDR<0.05; |Log2 fold-change ratio| >1.0) was used to select differentially expressed genes. The final differentially expressed gene list was selected as the intersection between the comparisons against the two controls: αD11 vs. antiV5 and αD11 vs. naive. Gene Ontology analysis was performed on differentially expressed upregulated and downregulated genes using DAVID (https://david.ncifcrf.gov/list.jsp). The enrichment score for the A1 (neurotoxic) or A2 (neuroprotective) expression phenotypes (according to Liddelow et al., 2017) was computed on the whole filtered dataset using the Gene Set Enrichment Analysis tool (GSEA, https://www.gsea-msigdb.org/gsea/index.jsp). PCA was computed using the *scikit-learn* package in Python. Dataset normalization was performed using the function *StandardScaler*: each gene variable was independently mean-centered and scaled to unit variance.

### 2.8 Primary neuronal cultures and neuron–astrocyte co-cultures

To derive primary hippocampal neurons, we used a modified protocol of that published by Beaudoin et al. (2012). Briefly, postnatal day 0 animals were decapitated, the brain was quickly dissected and placed in ice-cold Hank’s balanced salt solution (HBSS) (Thermo Fisher Scientific, MA, United States; #14180046), and the hippocampi were processed for 15 min at 37°C in DMEM-F12 containing 0.1% of trypsin (Thermo Fisher Scientific, MA, United States). The brain tissue was then gently disrupted using a pipette and plated in fresh DMEM containing 1% GlutaMAX, 10% FBS, 2% B27 supplement (Gibco, MA, United States, #17504044), 6 mg/ml glucose, 12.5 uM glutamate, and 10 ug/ml gentamicin (Gibco, MA, United States, #15710-049) at a density of 1.5 × 10^5^ cells in poly-D-lysine coverslips (Sigma Aldrich, MO, United States, #P1024). The cells were kept at 37°C in 5% CO_2_. After 24 h, pre-warmed neurobasal A medium (Thermo Fisher Scientific, MA, United States #10888-022) containing 2% of B27 supplement, 2.5 uM GlutaMAX, and 10 ug/ml gentamicin was added to the culture. At DIV (days *in vitro*) 17–19, primary astrocytes were seeded on the top of cultured hippocampal neurons (1 × 10^5^ cells/well) and allowed to rest for 24 h before starting the experiments.

#### 2.8.1 Immunocytochemistry on neuron–astrocyte co-cultures

Coverslips containing the co-cultures were first fixed in 2% PFA and 5% sucrose (10 min), then washed in PBS, and blocked for 1 h at RT in BSA 1%. Staining with primary antibodies rabbit anti-GFAP (DakoCytomation, Glostrup, Denmark, Z0334, 1: 500) or goat anti-GFAP (Santa Cruz Biotechnology, California, United States of America, sc-6170, 1:300), anti-MAP2 antibody (Abcam, ab92434; 1:500), and anti-caspase 3 antibody, active (cleaved) form (AB3623, Sigma-Aldrich; 1:100) was performed overnight at 4°C. Fluoroshield (Sigma Aldrich, MO, United States #F6057) containing DAPI was used to mount the coverslip, and a Leica SP2 confocal microscope (Leica Microsystems, Wetzlar, Germany) equipped with an HCX PL APO 63.0X OIL (NA = 1.40) objective was used to acquire images.

#### 2.8.2 Morphological analysis of neurons

Hippocampal astrocytes and neurons were first cultured separately (for 2 weeks and 1 week, respectively) to ensure culture homogeneity and purity. At the end of the pre-incubation period, the individual cultures were mixed at a 1:1 ratio by detaching astrocytes from their dish and plating them onto the neuronal culture. To evaluate neuronal health, we measured morphological alterations commonly used to determine degenerating cells (Ertürk et al., 2014). MAP2 immunoreactivity was adopted to label neurons, and the following parameters were used to define neuronal health: i) degeneration of soma, with or without detached dendrites, ii) blebbing dendrites, or iii) dendrite deterioration and rupture. Image processing and analyses were carried out in Fiji/ImageJ.

#### 2.8.3 Calcium imaging in neurons *in vitro*

Oregon Green™ 488 BAPTA-2 (O6809, Thermo Fisher) (1 µM) was used as the calcium indicator to detect calcium transients in neurons. Neurons were plated in an imaging well at DIV 0 and kept until DIV 14. Astrocytes were then added to the culture at a density of 1 × 10^5^ cells/well. After 24 h, co-cultures were treated with the calcium indicator for 20 min and then washed carefully as specified in the datasheet. Analysis was performed similarly to the procedure mentioned in *2.6.2 Calcium imaging in astrocytes in vitro.* Neurons were identified from astrocytes after recording by MAP2 and GFAP differential labeling and comparison to the calcium-recorded image.

### 2.9 *In vivo* two-photon microscopy

#### 2.9.1 AAV injections

The genetically encoded calcium sensor GCaMP6f was expressed with recombinant AAV under the human GFAP promoter (gfaABC1D-cyto-GCaMP6f, Addgene). Then, 0.2 µl of AAV virus was injected (Picospritzer III; 20 p. s.i., 20 m, 0.2 Hz) into layer I of the primary motor cortex (coordinates: 300 µm anterior and 1,500 µm lateral to bregma, depth of 50 µm). A micromanipulator (M3301 World Precision Instruments) was used to insert the glass pipette into the brain at an angle of 60°. Injections were performed on postnatal day 21, while imaging was carried out 30 days later.

#### 2.9.2 Two-photon calcium imaging in the anesthetized mouse

The small cranial window imaging technique was performed to image GCaMP6f-expressing astrocytes, as previously published (Grutzendler et al., 2002). Animals were anesthetized intraperitoneally with ketamine (200 mg/kg) and xylazine (30 mg/kg) diluted in saline. A region 1–2 mm in diameter was first thinned and then opened using forceps. The dura mater was also removed to allow drug penetration. The brain surface was kept under artificial mouse cerebrospinal fluid (ACSF, 125 mM NaCl, 26 mM NaHCO_3_, 1.25 mM NaH_2_PO_4_, 2.5 mM KCl, 1.0 mM MgCl_2_, 2.0 mM CaCl2, 25 mM glucose; bubble flow 95% O_2_ and 5% CO_2_ through the solution) at all times. The skull was attached to a custom-made steel plate to reduce movement during the imaging sessions. For imaging, either an Olympus multi-photon microscope (FV1000) or an Ultima Investigator two-photon microscope (Bruker) was used. The laser of the microscope (Ti-sapphire) was tuned to 920 nm to match the excitation spectrum of GCaMP. Recordings consisted of time-lapses at 4 Hz acquired using a water-immersion objective (either Olympus ×60, 0.9 N.A. or Olympus 25X, N.A. 1.1) at an optical zoom range of 1.0–3.0. The fields acquired were at a depth of ∼200 µm from the pial surface.

#### 2.9.3 Surgery and imaging in awake mice

The procedure for imaging awake head-restrained mice was performed 24 h prior to imaging in order to attach a head-restraining device (Yang et al., 2013). Briefly, the mice were anesthetized with an injection of ketamine (100 mg/g) and xylazine (10 mg/g). The head-restraining device, meant to reduce motion artifacts, consisted of two parallel metal bars secured to the animal’s skull using dental acrylic. The next day, we created a cranial window using a similar method to the procedure mentioned in 2.9.2. Before imaging, the mice were habituated to the imaging apparatus for 10 min each to diminish possible stress due to head restraining. Imaging consisted of 120-second recording sessions in which the animal was made to run for 80 s in total.

#### 2.9.4 Drug application through craniotomy

Drugs were dissolved in ACSF, and 200 μl of the solution was applied directly onto the cranial window during craniotomy (for awake-state experiments) or under the two-photon microscope (in the anesthesia experiments). We used the following drugs: NGF (1 μg/ml; Alomone labs), phenylephrine (Sigma, 100 nM), antiNGF clone αD11 (8 μg/ml, purified in-house from the hybridoma cell line supernatant), and TrkAFc (1 μg/ml, R&D Systems). The NT condition in all *in vivo* experiments refers to vehicle/ACSF.

#### 2.9.5 Data analysis of GCaMP6 signal

Visually identifiable processes or somata were selected for quantification. Stacks acquired were first registered using the ImageJ plugin StackReg to minimize movement on the x–y plane. The fluorescence time course of each soma or process identified in the recording was extracted using ROI Manager in ImageJ software, obtaining the average of all pixels within the selected regions of interest at each time point. The ΔF/F0 value was calculated as ΔF/F0 = (F − F0)/F0 × 100%, in which F0 is the baseline fluorescence signal averaged over a 2 s period before observing calcium activity or the administration of PE or during the 20 s of baseline (for awake-state experiments). A region adjacent to the astrocytes was used as the background.

### 2.10 Statistical analyses

All data in this work are presented as mean ± SEM. Statistical analysis was performed using GraphPad Prism. Comparison between the two groups was performed using the two-tailed Student’s t-test when data passed normality (Kolmogorov–Smirnov test) and equal variance (F-test) tests and using the Wilcoxon matched-pairs signed rank test and Mann–Whitney *U* test when they did not pass the normality test. One-way ANOVA and two-way ANOVA were used when confronting groups n > 2 and two different factors, respectively.

## 3 Results

### 3.1 Asthenic astrocyte morphology *in vivo* in different NGF/proNGF unbalance transgenic models

**FIGURE 1:**
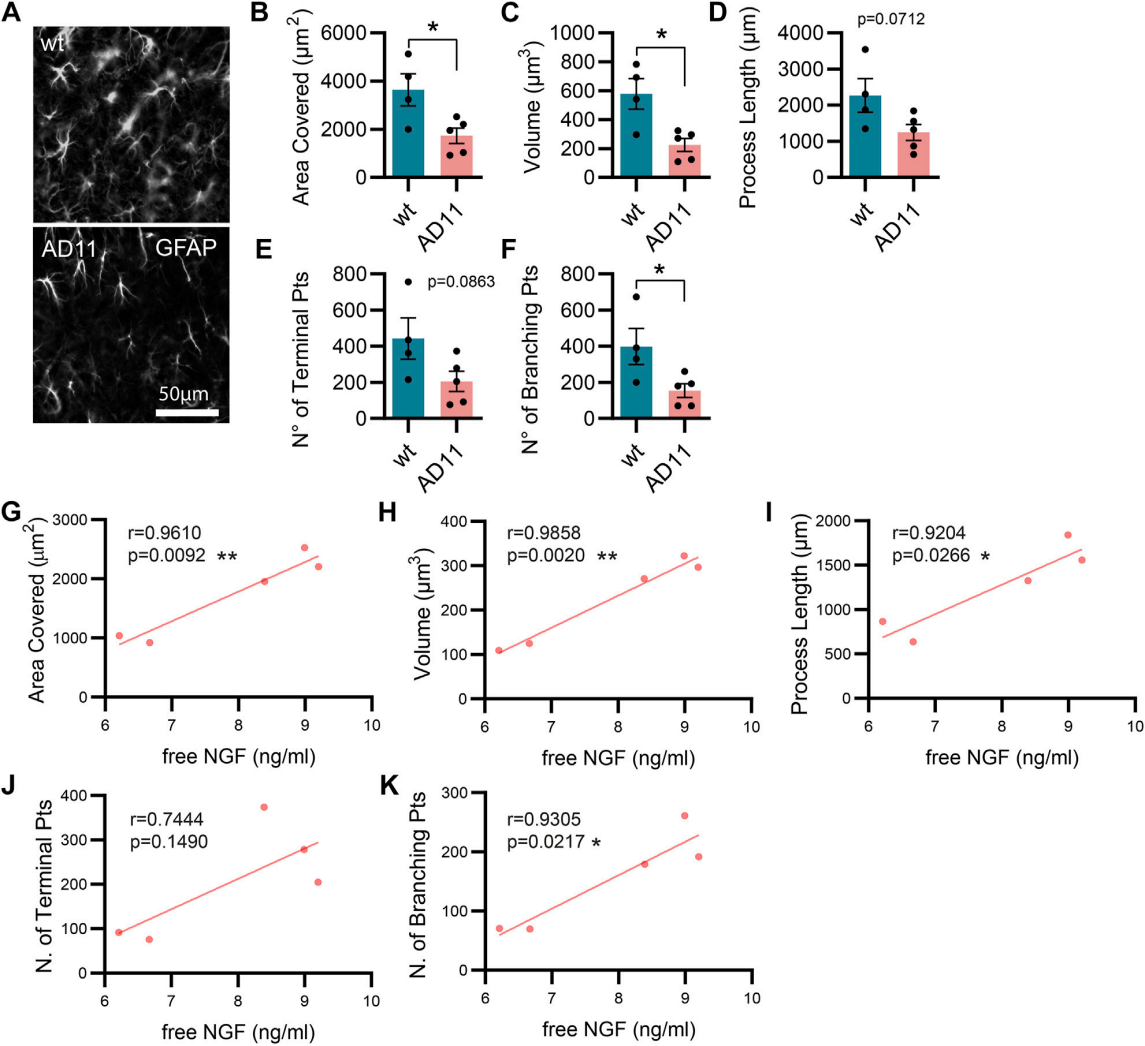
Alterations in astrocyte morphology *in vivo* in AD11 antiNGF transgenic mice. **(A)** Representative images of GFAP-labeled astrocytes and their 3D reconstruction using Imaris from 2-month-old AD11 and control mice (*n* = 4 WT, *n* = 5 AD11 mice, four–five cells per animal). **(B)** Barplot representing the area covered by astrocyte processes in control and AD11 mice (two-tailed unpaired *t*-test; *p* = 0.0278). **(C)** Barplot representing the total astrocyte volume in control and AD11 mice (two-tailed unpaired *t*-test; *p* = 0.0126). **(D)** Barplot representing the overall length of astrocytic processes in control and AD11 mice (two-tailed unpaired *t*-test; *p* = 0.0712). **(E)** Barplot representing the number of terminal points in control and AD11 mice (two-tailed unpaired *t*-test; *p* = 0.0863). **(F)** Barplot representing the number of branching points in control and AD11 mice (two-tailed unpaired *t*-test; *p* = 0.0404). **(G–K)** Pearson correlation between free NGF quantified by ELISA and all morphological parameters: **(G)** area covered, **(H)** total astrocyte volume, **(I)** length of astrocytic processes, **(J)** number of terminal points, and **(K)** number of branching points. Pearson coefficients and two-tailed *p*-values are reported in the figures.

**FIGURE 2:**
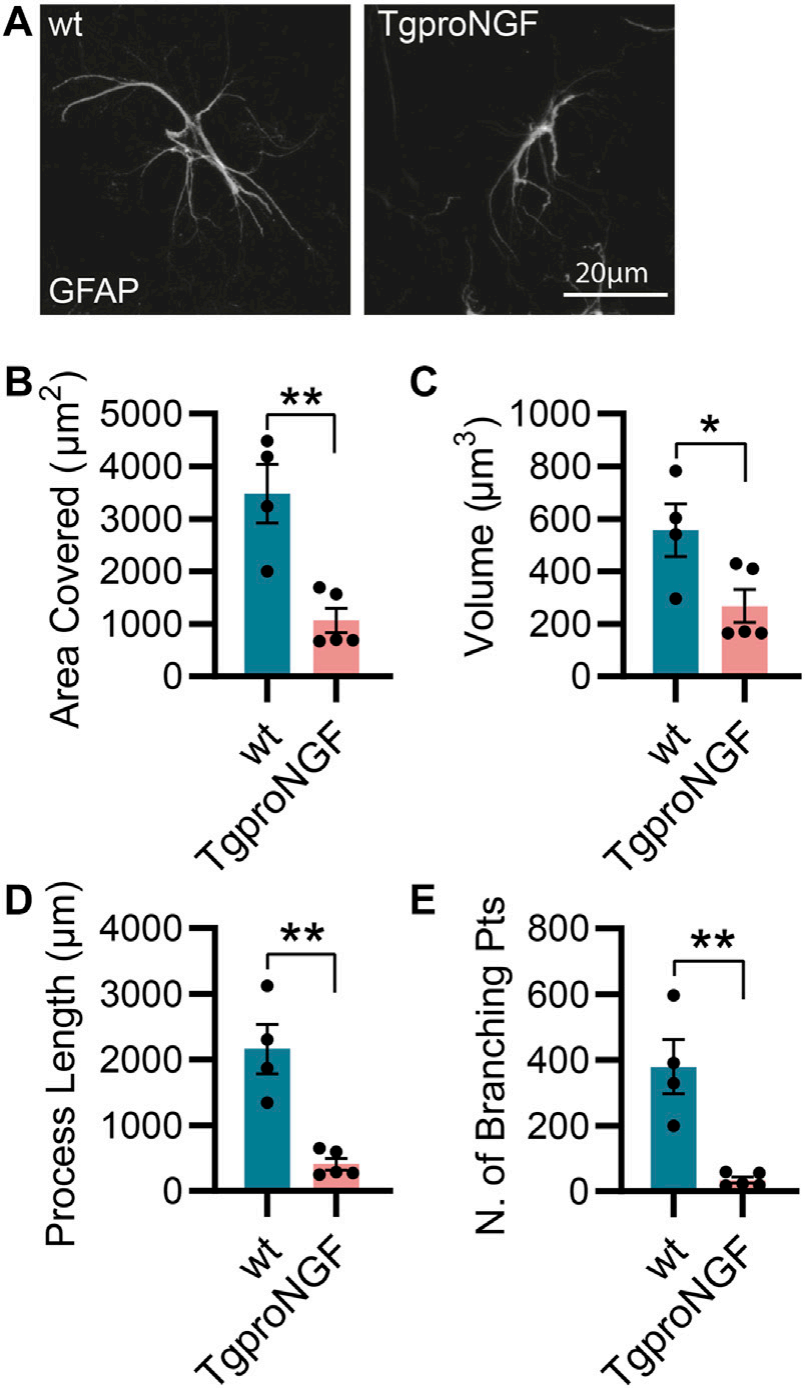
Alterations in astrocyte morphology *in vivo* in 2-month-old TgproNGF#72 mice. **(A)** Representative images of astrocytes and their 3D reconstruction using Imaris from 2-month-old TgproNGF#72 and control mice (*n* = 4 control mice, *n* = 5 in TgproNGF#72 mice; four–five cells per animal). **(B)** Barplot representing the area covered by astrocyte processes in control and TgproNGF#72 mice (two-tailed unpaired *t*-test; *p* = 0.0035). **(C)** Barplot representing the astrocytic volume in control and TgproNGF#72 mice (two-tailed unpaired *t*-test; *p* = 0.0379). **(D)** Barplot representing the process length in control and TgproNGF#72 mice (two-tailed unpaired *t*-test; *p* = 0.0014). **(E)** Barplot representing the number of branching points in control and TgproNGF#72 mice (two-tailed unpaired *t*-test; *p* = 0.0022).

As mentioned previously, the AD11 mouse model, constitutively expressing an antibody preferentially neutralizing mature NGF with respect to proNGF (mAb αD11), displays a progressive Alzheimer-like neurodegeneration (Capsoni et al., 2000; Ruberti et al., 2000). Similarly, the cleavage-resistant proNGF mouse (TgproNGF#72) develops memory impairments and cholinergic deficits reminiscent of AD mouse models (Tiveron et al., 2013). To understand the impact of modulating NGF/proNGF levels on astrocyte physiology, we used these two mouse models and analyzed astrocyte morphology in the hippocampus of 2-month-old AD11 and TgproNGF#72 mice. We found that in AD11 mice, before the start of overt neurodegeneration (Capsoni et al., 2000; Capsoni et al., 2011), GFAP-labeled astrocytes display a striking morphological phenotype, with reduced process arborization compared to control mice, specifically in the length of the processes, number of branching points, total area covered by the astrocytic processes, and overall volume (Figures 1A–E). In the AD11 model, neutralization of NGF by the transgenic antibody leads to variable amounts of captured NGF. To understand whether this variability impacted astrocyte morphology, we quantified free NGF in whole-brain homogenates of individual AD11 mice *via* ELISA. The amount of NGF was shown to correlate very precisely with four out of five parameters of astrocyte morphology (Figures 1G–K), suggesting NGF directly modulated astrocyte morphology. An even more pronounced morphological phenotype of reduced complexity is encountered in the TgproNGF#72 mouse model of neurodegeneration at 2 months of age (Figures 2A–E). To further understand the effect of stunted NGF signaling on astrocytes, we analyzed their morphology in the p75^NTR−/−^ mice and in a transgenic mouse model lacking a copy of TrkA gene (heterozygous TrkA^+/−^ knock-out mice) (Supplementary Figure S1). Interestingly, we observed astrocytic atrophy in mice lacking p75^NTR^ signaling (Supplementary Figures S1A–E), but no effect was observed in TrkA^+/−^ mice (Supplementary Figures S1F–J), suggesting the importance of p75^NTR^ signaling for the homeostasis and physiological welfare of astrocytes.

In conclusion, interfering with NGF levels and signaling leads to altered astrocyte morphology in multiple mouse models.

### 3.2 The neutralization of NGF in primary hippocampal astrocyte cultures affects their morphology

**FIGURE 3:**
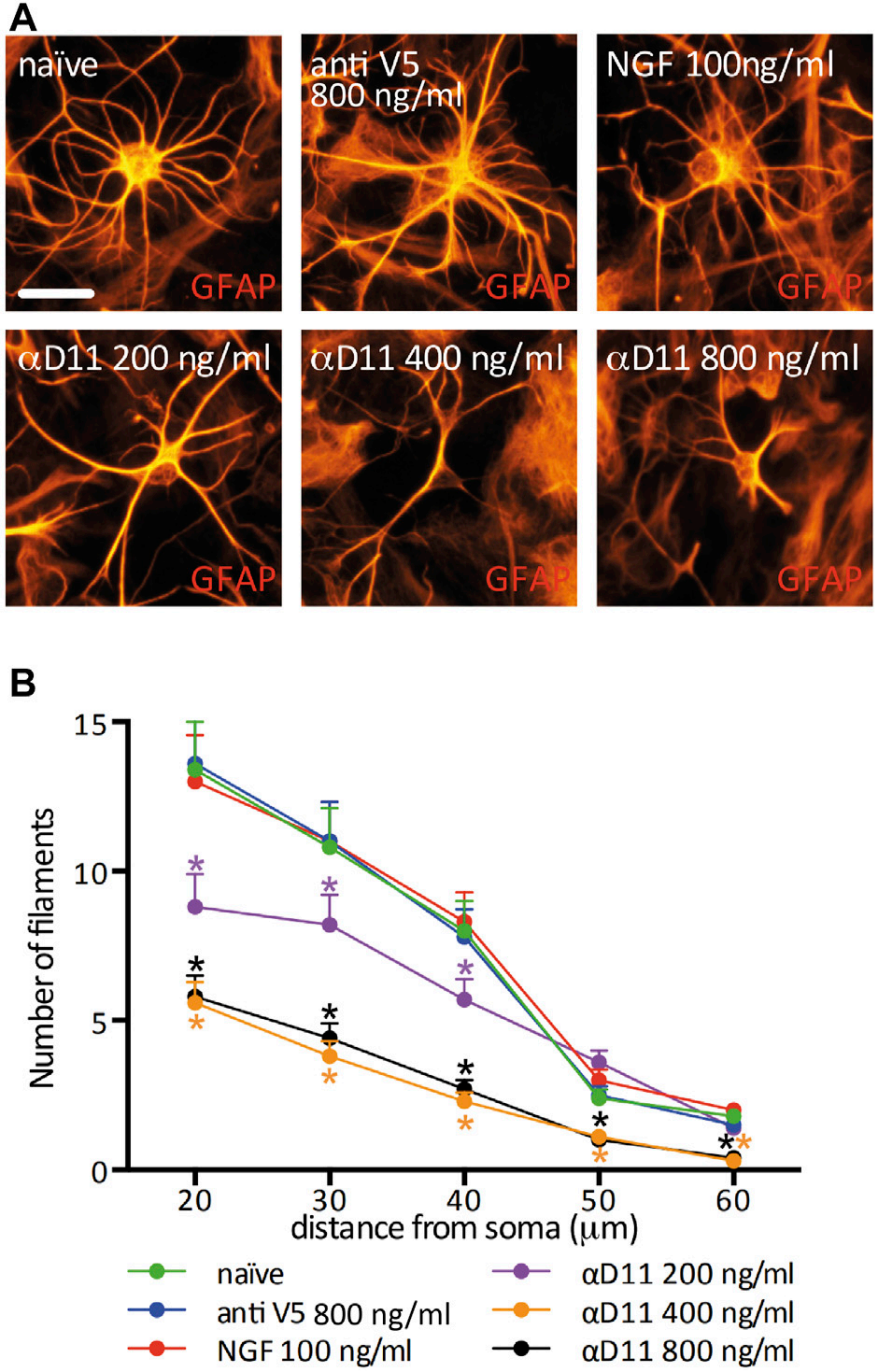
Astrocyte morphology is highly affected by the lack of mature NGF *in vitro*. **(A)** GFAP-labeled primary astrocytes after a 48 h treatment with NGF, antiV5 (non-relevant control antibody), or αD11 antibody. **(B)** Sholl analysis of astrocyte morphology (RM One-way ANOVA: *p* < 0.05; Dunnett’s multiple comparison test; asterisks refer to the comparison between the naive and the treatments at each distance).

Having established that interfering with NGF signaling in the mouse brain induces an asthenic morphological phenotype of astrocytes, we investigated whether the mechanism involved is cell-autonomous. To this aim, we considered an *in vitro* condition and analyzed the response of primary hippocampal astrocytes to the modulations of NGF levels. Astrocytes express the NGF receptor p75^NTR^ *in vitro* and *in vivo* (Hutton et al., 1992; Cragnolini et al., 2009; Vignoli et al., 2016) and, to a certain extent, TrkA (Connor et al., 1996; Aguado et al., 1998; Oderfeld-Nowak et al., 2001; 2003; Capsoni et al., 2017). Moreover, astrocytes express and secrete NGF (Friedman et al., 1996; Domeniconi et al., 2007). In our culture conditions, we determined the concentration of total NGF (mature NGF and proNGF) by ELISA (Supplementary Figures S2A, B) to be in the range of 25–50 pg/ml. In principle, then, cultured astrocytes can support an autocrine or a paracrine NGF signaling loop, making this culture system a proper model to investigate the consequences of interrupting such a loop in well-defined conditions. Thus, we treated hippocampal primary astrocytes for 48 h either with mNGF (100 ng/ml) or with increasing doses of the antiNGF antibody mAb αD11 (αD11). As a control for αD11, we used both the vehicle condition (naive) and a non-relevant antibody that recognizes a synthetic peptide, mAbantiSV5 (antiV5). We observed that astrocyte morphological complexity was unaltered by administering mNGF or antiV5 (Figures 3A, B). On the other hand, NGF neutralization *via* αD11 caused a dose-dependent (200–800 ng/ml) progressive astrocytic atrophy. In conclusion, depriving astrocytes of NGF signaling affects astrocyte morphology *in vitro*.

### 3.3 NGF neutralization increases astrocyte calcium activity *in vitro*

**FIGURE 4:**
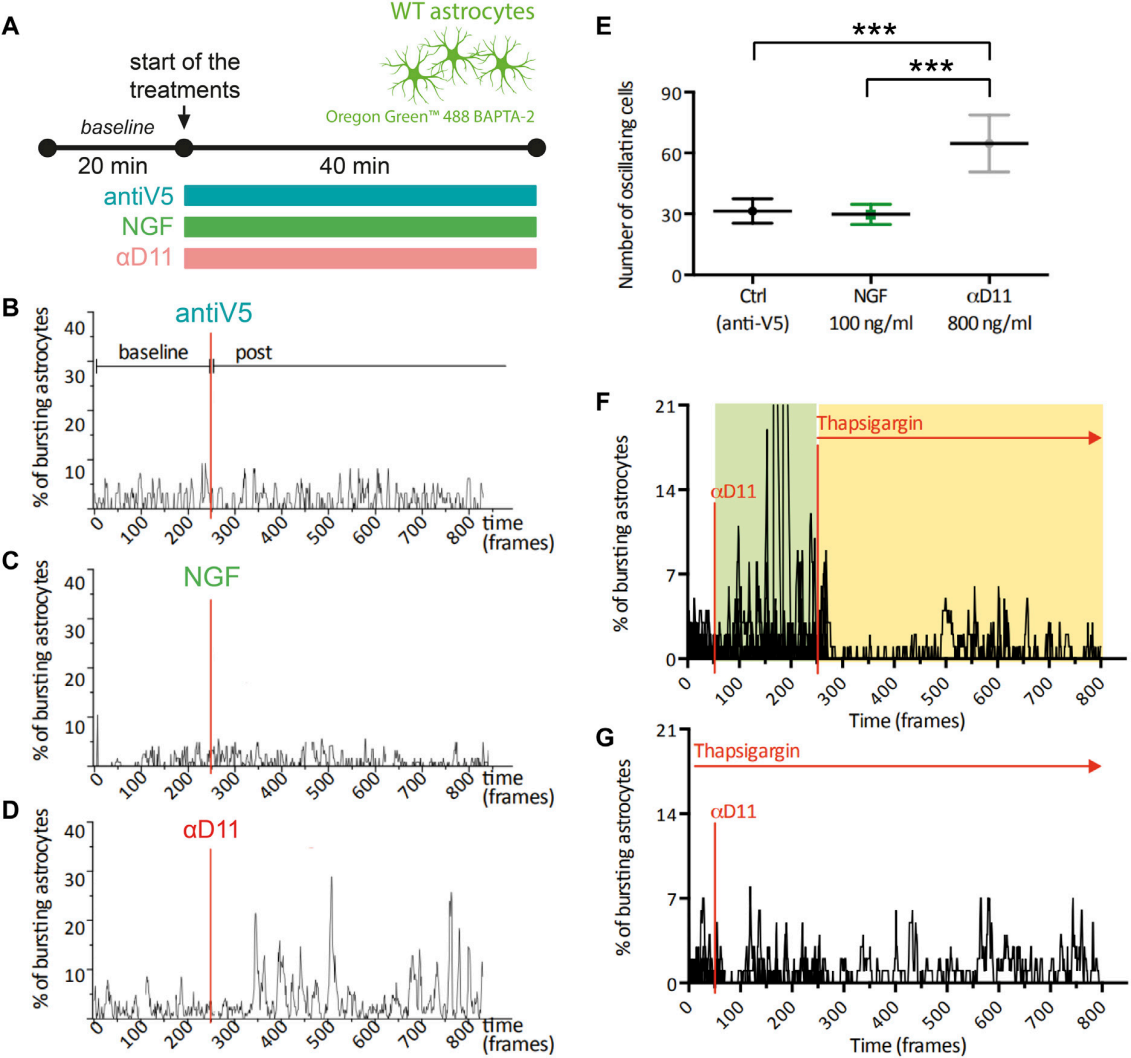
Calcium response of cultured astrocytes to NGF, a neutralizing antibody for NGF (αD11), and a control antibody (antiV5). **(A)** Timeline of the imaging experiment: primary astrocytes were loaded with Oregon 488 BAPTA-2 and recorded up to 1 h. First, a baseline was acquired, and then cultures were treated with either the control antibody (antiV5), NGF, or αD11. **(B–D)** Representative traces and **(E)** means of the percentages of responsive astrocytes during different treatments (One-way ANOVA, ctrl vs. NGF n.s., ctrl vs. αD11 *p* < 0.001, ctrl vs. NGF *p* < 0.001). **(F, G)** Percentages of responding astrocytes treated with αD11 and thapsigargin. Thapsigargin was added either after **(F)** or before **(G)** αD11 addition.

One of the most functionally relevant responses in the spectrum of astrocyte activation is the generation of calcium (Ca^2+^) transients. These Ca^2+^ elevations have been connected to modulations at the synaptic level of neuronal computation and activity (Santello et al., 2019; Lia et al., 2021). To determine the functional response of astrocytes to changes in the level of extracellular NGF, we imaged primary hippocampal astrocytes previously stained with the Ca^2+^ indicator Oregon Green BAPTA 2 in the presence of mNGF (100 ng/ml), of αD11, or of the control antibody, antiV5 (Figure 4A). No change was observed in the spontaneous Ca^2+^ activity in response to either NGF or antiV5 (Figures 4B, C). On the other hand, astrocytes responded to NGF neutralization *via* αD11 (800 ng/ml) by dramatically increasing their Ca^2+^ transients, within 5 min from the start of αD11 treatment (Figures 4D, E). Ca^2+^ events can either be generated from Ca^2+^ release from intracellular stores *via* the IP3 receptors (IP3Rs) located in the endoplasmic reticulum (ER) or from the extracellular compartment *via* transmembrane Ca^2+^ channels like TRPA1 (Khakh and McCarthy, 2015). To determine the origin of the Ca^2+^ involved in αD11-mediated Ca^2+^ transients, we thus treated astrocytes with thapsigargin, an inhibitor of SERCA channels that transport Ca^2+^ from the cytosol to the ER, thereby depleting intracellular stores of Ca^2+^. Decreased astrocyte Ca^2+^ responses to αD11 were observed in the presence of thapsigargin, either when this drug was applied before or after αD11 administration, as qualitatively shown in Figures 4F, G. Thus, we conclude that lack of NGF induces Ca^2+^ release from intracellular stores in primary astrocytes.

### 3.4 Astrocytes respond to a reduced NGF signaling by turning on a reactive A1-transcriptional program

**FIGURE 5:**
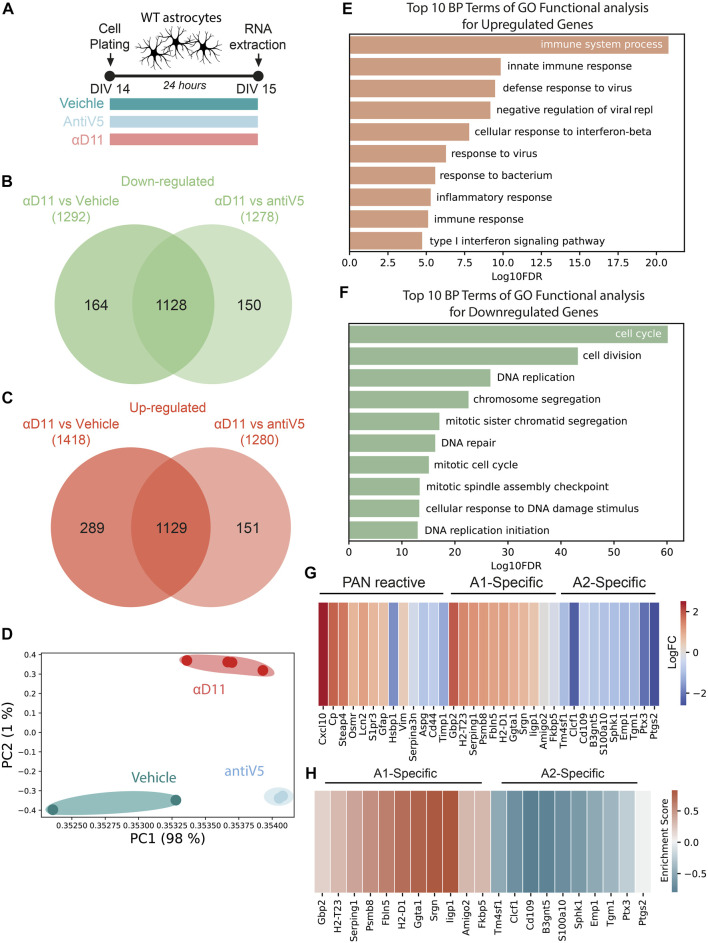
Astrocytes respond to a reduced NGF signaling by turning on a reactive A1-transcriptional program. **(A)** Timeline of the experiment: primary astrocytes were treated for 48 h with either vehicle, antiV5, or αD11. **(B, C)** Venn diagram of downregulated **(B)** and upregulated **(C)** genes by αD11. **(D)** PCA of the expression data. Each point represents a sample. **(E, F)** The 10 most significantly (FDR<0.05) enriched GO terms in *biological processes* for **(E)** upregulated and **(F)** downregulated genes performed using DAVID. **(G)** Heatmap of PAN reactive and A1- and A2-specific reactive transcripts following αD11 administration. Data are expressed as LogFC values. **(H)** Heatmap represents the enrichment scores resulting from the GSEA evaluating the enrichment of A1 or A2 phenotype-associated genes.

The aforestated results show that NGF neutralization has a profound effect on the morphology and physiology of astrocytes. To further understand these responses, we performed a transcriptomic analysis by Agilent microarray of primary astrocytes 24 h after αD11 administration (800 ng/ml) (Figure 5A). Overall, αD11 administration induced a profound change in gene expression in astrocytes: we found 1,128 differentially downregulated genes and 1,129 differentially upregulated genes in αD11-treated astrocytes compared to antiV5 and vehicle conditions (Figures 5B, C). Principal component analysis (PCA) highlighted that the differential transcriptomic profile induced in astrocytes by αD11 is very specific and well-distinct from control profiles (Figure 5D). Gene Ontology (GO) analysis of the differentially expressed mRNAs reveals a remarkably consistent pattern: the top 10 upregulated GO terms belong to immune-related pathways (Figure 5E), suggesting that αD11-treated astrocytes are proinflammatory and reactive. Conversely, the top downregulated GO terms list processes involved in cell cycle and cell replication (Figure 5F), which is in line with previous suggestions indicating the involvement of NGF signaling in astrocyte proliferation (Cragnolini et al., 2009; Cragnolini et al., 2012). Furthermore, StringDB analysis of the top 300 modulated genes independently showed that upregulated pathways are related to immune functions (Supplementary Figures S3, red cluster), while downregulated pathways show clusters of genes related to the cell cycle (Supplementary Figures S4, red cluster), and to extracellular matrix (Supplementary Figures S4, green cluster). The top 50 upregulated and downregulated mRNAs are listed in Supplementary Table S1. Previous studies have identified two different types of reactive astrocytes based on the transcriptional profiles acquired by these cells in mice that were either treated with systemic injection of lipopolysaccharide (LPS) or that received middle cerebral artery occlusion to induce ischemia: neurotoxic (A1) astrocytes that can cause neuronal death and neuroprotective (A2) astrocytes that promote neuronal survival and tissue repair (Zamanian et al., 2012; Liddelow et al., 2017; Guttenplan et al., 2021). A set of genes commonly modulated in both types of astrocyte treatment (PAN reactive gene list) provide a common fingerprint for reactive astrocytes (Liddelow et al., 2017).

We thus first looked at the expression of A1- (neurotoxic) and A2-specific (neuroprotective) transcripts in our dataset and discovered that antiNGF-treated astrocytes upregulated all the top 10 A1-specific mRNAs and downregulated all the top 10 A2-specific genes (Figure 5G). On the other hand, the PAN reactive genes show a mixed profile (Figure 5G). To further corroborate the data, we then performed a Gene Set Enrichment Analysis (GSEA) and looked for the expression of genes characterizing the A1 (neurotoxic) or A2 (neuroprotective) fingerprint profile. We observed that our dataset shows enrichment for A1 genes (FDR = 0.001) and a negative correlation with A2 genes (FDR<0.001), suggesting that a 24 h administration of αD11 steers astrocytes toward a neurotoxic A1 phenotype (Figure 5H). Conversely, no enrichment was observed for the PAN reactive gene list (FDR = 0.202). Overall, these results show that reducing NGF levels profoundly affects astrocytes, steering them toward a neurotoxic A1-phenotype.

### 3.5 NGF-deprived astrocytes acquire a neurotoxic phenotype: Neuron–astrocyte co-culture experiments

**FIGURE 6:**
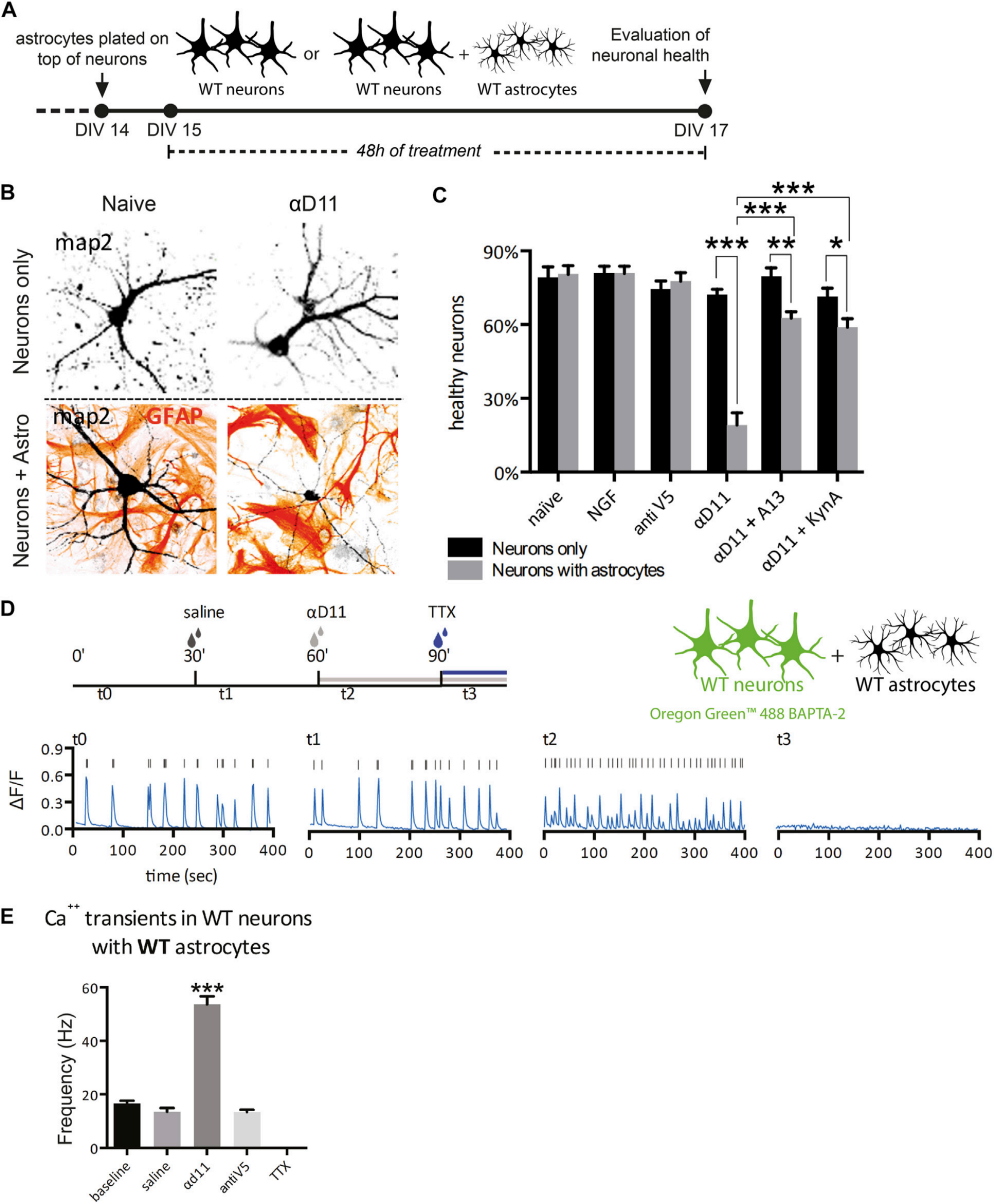
NGF-deprived astrocytes are neurotoxic: neuron–astrocyte co-culture experiments. **(A)** Timeline of the experiment: neurons or neuron–astrocyte co-cultures were treated for 48 h with either vehicle, NGF, antiV5, αD11, αD11 plus the anti-amyloid antibody A13, or αD11 plus kynurenic acid. **(B)** Representative images of neuron–astrocyte co-cultures in the presence of the NGF-neutralizing antibody αD11. **(C)** Quantification of the percentage of healthy neurons in the different experimental conditions (two-way ANOVA with Sidak’s multiple comparison test, *p* < 0.05*, *p* < 0.01**, and *p* < 0.001***). **(D)** Timeline of the calcium imaging experiments in neurons. First, neuron–astrocyte co-cultures were acquired in baseline condition (t0). Then, vehicle (saline) was added (t1). Later, the same co-culture was treated with αD11 (t2) (or with negative control antiV5 in parallel) and then with TTX (t3). **(E)** Quantification of the firing frequency in response to the different treatments (One-way ANOVA, Dunnett’s multiple comparison test; baseline vs. αd11: *p* < 0.001).

Having established that primary astrocytes respond to reduced NGF signaling by turning on a reactive A1 transcriptional program, we experimentally investigated the predicted neurotoxicity of NGF-deprived astrocytes. To this aim, we cultured hippocampal astrocytes together with neurons and assessed the neuronal health after NGF deprivation (Figures 6A, B). We treated, for 48 h, either neurons alone or astrocyte–neuron co-cultures with 1) NGF (100 ng/ml), 2) non-relevant antibody antiV5 (as a negative control, 800 ng/ml), 3) αD11 (800 ng/ml), or 4) vehicle. At the end of the treatments, we evaluated neuronal health and wellbeing by assessing morphological modifications frequently found in degenerating or dying neurons (Ertürk et al., 2014). Of note, the percentage of healthy neurons cultured alone is comparable to that of neurons co-cultured with astrocytes in naive condition (vehicle) and neurons co-cultured with astrocytes treated with NGF or antiV5 (Figure 6C). On the other hand, αD11 caused a robust decrease in the percentage of healthy neurons, but only in neurons co-cultured with astrocytes, suggesting astrocytes mediate the neurotoxic effect of NGF deprivation (Figure 6C). This correlates well with a measure of caspase-3 cleavage by immunofluorescence with a cleaved-caspase-3 antibody (see Methods) in co-cultures after treatment with vehicle, NGF, antiV5, and αD11. Only after αD11 treatment, the caspase-3 cleavage was found to increase significantly, up to 4-fold (Supplementary Figures S5).

It has been previously reported that treatment of NGF in hippocampal neurons leads to rapid activation of the amyloidogenic pathway and causes neuronal apoptotic death (Matrone et al., 2008). We investigated whether amyloid-β (Aβ) contributes to neuronal death driven by NGF-deprived neurotoxic astrocytes. To this aim, the antiNGF-treated astrocyte–neuron co-cultures were concomitantly incubated with scFv A13, a recombinant antibody domain specifically binding to oligomeric forms of the Aβ peptide, with great selectivity with respect to monomeric or fibrillar forms of Aβ (Meli et al., 2009; Meli et al., 2014). This concomitant treatment caused significant reduction of the αD11-driven neurotoxicity, effectively counteracting the loss of healthy neurons (Figure 6C), suggesting the involvement of oligomeric forms of Aβ peptide in driving the neurotoxic effects by antiNGF-treated astrocytes. Another well-known mechanism of astrocyte-driven neurotoxicity is excitotoxicity. In particular, astrocytes cause excitotoxicity *via* the release of gliotransmitters such as glutamate (Ding et al., 2007). To evaluate the contribution of excitotoxicity to αD11-driven neurotoxicity, we administered kynurenic acid (50 μM), an antagonist of the glutamate ionotropic excitatory amino acid receptors, concomitantly with αD11. This treatment partially reversed the effect of αD11 administration, suggesting the involvement of glutamate-mediated excitotoxicity in antiNGF-driven neurotoxicity (Figure 6C).

To further validate the neuronal excitotoxicity driven by NGF deprivation of astrocytes, we then researched whether NGF deprivation could trigger Ca^2+^ hyperactivation in neurons. For this purpose, neuron–astrocyte co-cultures were incubated with αD11, and Ca^2+^ transients were measured in the neuronal component of the co-culture (Figures 6D, E). First, neuronal Ca^2+^ transients were acquired in the baseline condition (t0), and then the vehicle was added (t1). Later, the same co-culture was treated with αD11 (t2) (or with negative control antiV5 in a parallel co-culture), and last with tetrodotoxin (TTX) (t3) (Figure 6D). Interestingly, αD11 significantly increased the frequency of neuronal Ca^2+^ transient events during the 400 s time period (Figures 6D, E). The antiNGF-induced neuronal Ca^2+^ signals were completely abolished by subsequent treatment with TTX, which is in line with their neuronal origin of that measured Ca^2+^ signal (Figure 6E). In conclusion, these experiments with hippocampal astrocyte–neuron co-cultures *in vitro* demonstrate that NGF-deprived astrocytes acquire a neurotoxic phenotype. Activation of an amyloidogenic pathway with the production of Aβ oligomers and glutamate-dependent Ca^2+^ excitotoxicity is suggested to be part of the antiNGF-induced neurotoxic pathway.

### 3.6 Astrocytes respond to the lack of extracellular NGF increasing calcium activity *in vivo*

**FIGURE 7:**
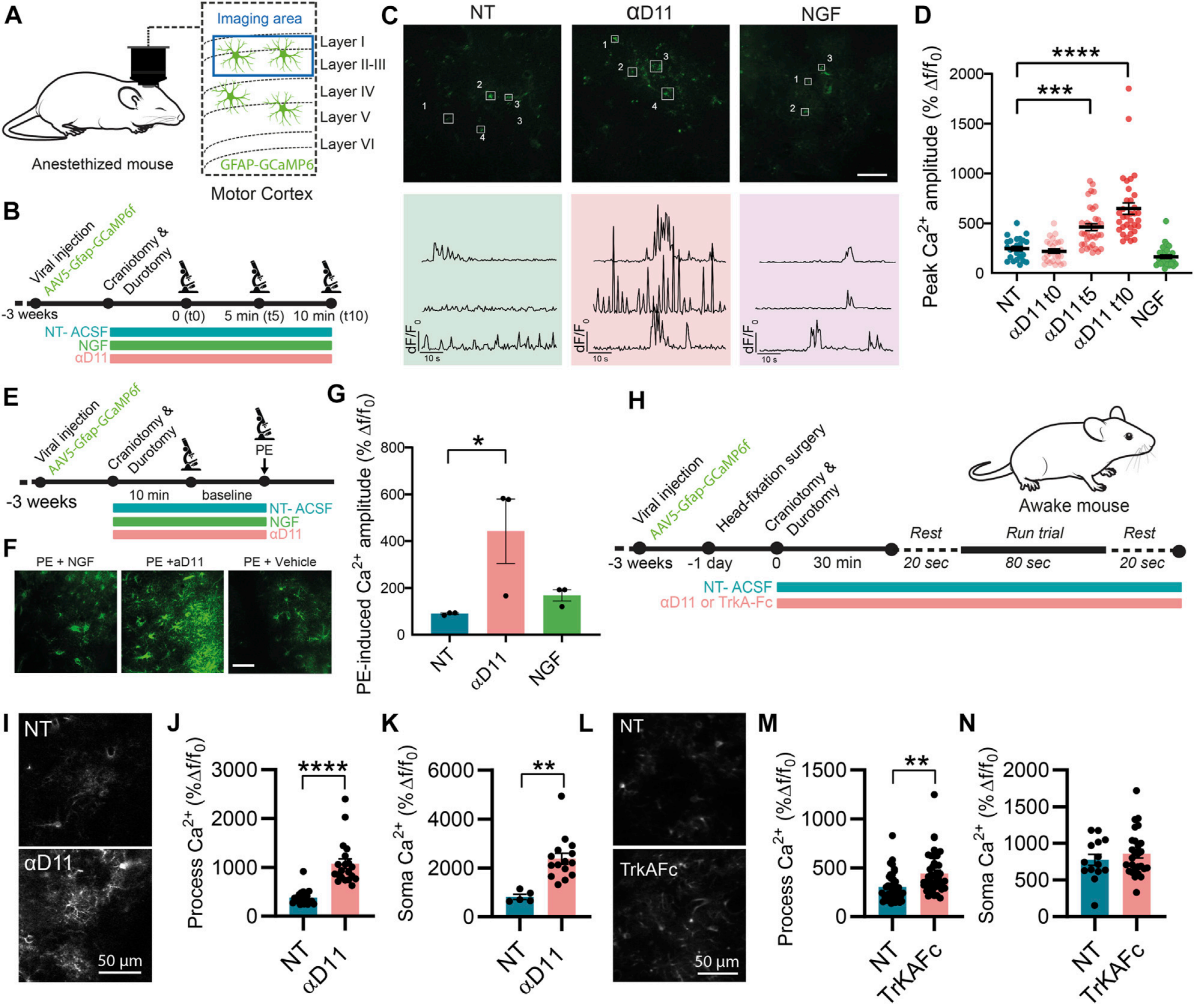
Astrocytes respond to the lack of extracellular NGF increasing calcium activity *in vivo*. **(A)** Imaging was performed in layer I of the motor cortex of anesthetized mice expressing GCaMP6f in astrocytes. **(B)** Timeline of the experiment recording astrocyte activity. **(C)** Representative two-photon images and traces of Ca^2+^ response in astrocytic processes expressing GCaMP6f before (NT) and after NGF (1 μg/ml) or αD11 (8 μg/ml) application in the motor cortex of an anesthetized mouse. **(D)** Quantification of the peak amplitude of Ca^2+^ transients in astrocytic processes during baseline (NT), right after (αD11 t0), after 5 min (αD11 t5), and after 10 min (αD11 t10) of αD11 (8 μg/ml) application (One-way ANOVA, NT vs. αD11 t5: *p* < 0.0001; NT vs. αD11 t10: *p* < 0.0001, n = 4 animals per group, ∼20 processes per animal). **(E)** Timeline of the experiment recording astrocyte activity during phenylephrine (PE) administration. **(F)** Representative images of non-treated (NT-ACSF), αD11-treated, and NGF-treated astrocytic response to PE 100 μM. **(G)** Quantification of the astrocytic response to PE 100 μM (One-way ANOVA, NT vs. αD11, *p* = 0.0001; NT vs. NGF, n.s.; NT αD11 vs. NGF, *p* = 0.0001; n = 3 animals per group, 10 cells per animal). **(H)** Timeline of the experiment recording astrocyte activity in awake-behaving animals: during the recording session, animals were made to run on a treadmill after 20 s of baseline. Astrocyte Ca^2+^ activity was recorded either **(I–K)** after administering αD11 or **(L, M)** another NGF neutralizing antibody (TrkA-Fc). **(I)** Representative images of the astrocytic responses. **(J)** Process peak amplitude (two-tailed unpaired *t*-test, *p* < 0.0001) to running in NT and αD11 treated conditions (n = 4 per group, ∼10 ROI per animal). **(K)** Soma peak amplitude (two-tailed unpaired *t*-test, *p* = 0.0012) to running in NT and αD11-treated conditions. **(L)** Representative images of the astrocytic response to TrkAFc. **(M)** Process peak amplitudes (two-tailed unpaired *t*-test, *p* = 0.001; n = 3 per group, ∼10 cells per animal). **(N)** Soma peak amplitudes (two-tailed unpaired *t*-test, n.s.) of the astrocytic Ca^2+^.

*In vitro* work is biased by differences in the activation state of primary astrocytes (Guttenplan and Liddelow, 2019). We then studied whether and how modulation of extracellular NGF levels affects astrocytes in their natural *in situ* environment—the brain. To do so, we performed two-photon calcium imaging *in vivo* in GCaMP6f-infected astrocytes in the motor cortex of anesthetized WT mice and quantified the acute response of these glial cells to the positive or negative modulations of NGF levels by applying NGF or the antiNGF neutralizing antibody αD11 (NGF 1 μg/ml, αD11 8 μg/ml) through a small cranial window (Figure 7A). The concentration of antiNGF antibody used is likely sufficiently high to saturate both mature NGF and proNGF proteins in the brain parenchyma. Astrocytic Ca^2+^ responses were monitored at three different timepoints: right after drug application (t0), after 5 (t5), and after 10 min (t10). Baseline Ca^2+^ activity did not change after NGF application at any time-point—data were thus pooled together (Figures 7B–D). Conversely, antiNGF αD11 treatment significantly increased astrocytic Ca^2+^ responses in astrocytic processes (Figure 7D). We then assessed the bulk activation of astrocytes, pretreated with NGF or with αD11 for 10 min, to the α1-receptor agonist phenylephrine (PE; 100 µM) (Figures 7E–G) in order to detect the global activation capacity of these glial cells (Ding et al., 2007). PE treatment elicited a much larger astrocytic Ca^2+^ response in αD11-treated mice compared with non-treated or NGF-treated cohorts (Figure 7G).

Since astrocytes responded strongly to lack of NGF in the cortex, we then evaluated the effect of NGF deprivation in the more complex system of the awake-behaving animals. We thus imaged GCaMP6f-expressing astrocytes in the mouse motor cortex of animals performing a running task, which is known to elicit strong Ca^2+^ responses both in the soma and fine processes of astrocytes (Paukert et al., 2014) (Figure 7H). We thus measured astrocyte Ca^2+^ responses under the influence of αD11 or not-treated (NT) conditions (Figures 7I–K). We report that, in a similar manner to the anesthetized condition, NGF deprivation *via* αD11 increases astrocytic activity both in the processes (Figures 7I, J) and soma (Figure 7K) in an animal performing a motor task. Similar results were obtained using another NGF inhibitor, TrkAFc (Figure 7L–N). In particular, TrkAFc could consistently increase astrocyte Ca^2+^ in processes (Figure 7M), though not in the soma (Figure 7N). In conclusion, using two different ways to decrease NGF levels in cortical tissue, we report that lack of NGF can increase acutely astrocyte Ca^2+^ *in vivo*. Altogether, these *in vivo* results extend the *in vitro* observations, further supporting the hypothesis that astrocytes can detect extracellular levels of NGF.

### 3.7 In neurodegenerative conditions, astrocytes acquire the ability to respond to exogenous NGF, decreasing calcium activity *in vivo*

**FIGURE 8:**
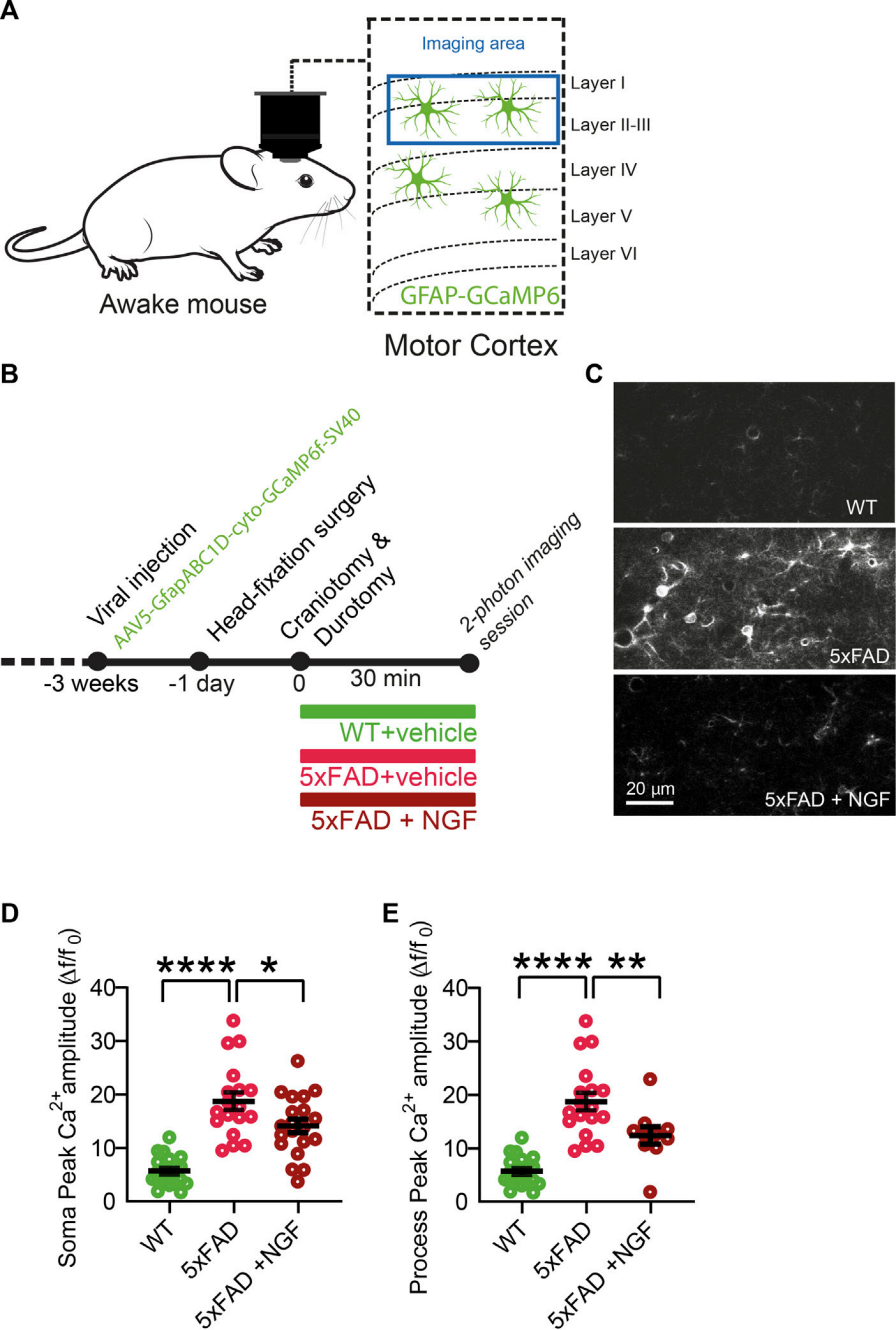
In neurodegenerative conditions (cortex of 5xFAD mice), astrocytes acquire the ability to respond to exogenous NGF by decreasing Ca^2+^ activity *in vivo*. **(A)** Imaging of astrocyte Ca^2+^ in 4-month-old WT and 5XFAD mice was performed in awake mice over the motor cortex. **(B)** Timeline of the experiment. **(C)** Representative two-photon images of astrocytic activity in WT and 5xFAD before and after NGF (1 μg/ml) administration. **(D)** Soma peak amplitudes (One-way ANOVA, WT vs. 5xFAD, *p* < 0.0001; WT vs. 5xFAD + NGF, *p* < 0.0001; 5xFAD vs. 5xFAD + NGF, *p* = 0.0262) of the astrocytic Ca^2+^. **(E)** (One-way ANOVA, WT vs. 5xFAD, *p* < 0.0001; WT vs. 5xFAD + NGF, *p* = 0.0044; 5xFAD vs. 5xFAD + NGF, *p* = 0.0083) of the astrocytic Ca^2+^ (*n* = 20 cells from three animals per group).

The results described previously demonstrate that astrocytes are very sensitive and react strongly to variations in the extracellular concentration of NGF. Very surprisingly though, we found that the responses of astrocytes to the modulation of NGF signaling are asymmetric, in that they only responded when the signaling was reduced, both in cultured astrocytes and *in vivo*. On the contrary, no response to added exogenous NGF could be demonstrated, possibly because in physiological conditions, the set point of the NGF-to-astrocyte signaling system is homeostatically saturated, highlighting a ceiling effect. We reasoned, therefore, that there might be some pathological situations, possibly linked to neurodegeneration, in which astrocytes may become sensitive to NGF, due to changes in the expression levels of p75^NTR^ and TrkA (Capsoni et al., 2017). We thus evaluated the effect of NGF on astrocyte Ca^2+^ responses *in vivo* in the 5xFAD mouse model of AD (Figures 8A, B), in which neurodegeneration is caused by mutations in Alzheimer’s-related genes and not by primary NGF deficits. First, we observed an increase in the astrocytic Ca^2+^ response in the 5xFAD at 4 months of age, with respect to age-matched WT mice (Figures 8D, E). Interestingly, though, when we applied NGF, the neurotrophin could significantly reduce astrocyte Ca^2+^, both in the soma and in the processes of cortical astrocytes (Figures 8D, E). Thus, in neurodegeneration, in which astrocyte Ca^2+^ responses are known to be excessive (Kuchibhotla et al., 2009), administering mature NGF can somehow dampen the excessive reactivity of astrocytes. It remains to be seen, in this *in vivo* situation, whether this effect of decreased astrocytes Ca^2+^ is a direct one or whether it is mediated by other NGF-target cells, such as microglia (Rizzi et al., 2018) or cholinergic terminals that innervate the cortex.

In order to investigate the potential NGF responsiveness of astrocyte asthenia in a neurodegenerative context, we considered 3xTG mice displaying a clear asthenic astrocyte phenotype until 5 months of age (Polis et al., 2018). We treated 3xTG mice between 1.5 and 2 months of age with intranasal NGF and evaluated astrocyte morphology after 2 weeks of treatment (Supplementary Figures S6A). It is well-established that this delivery route leads to a broad biodistribution of NGF in the brain, including the hippocampus (Capsoni et al., 2017). The morphology of astrocytes was evaluated by cell parameters such as volume, area, number of branching points, and length of the filaments on GFAP-labeled hippocampal slices, as shown in Figures 1, 2. We observed a robust and statistically significant increase in process length following treatment with NGF, compared to the administration of PBS, and a tendency of increase in the number of branching points (Supplementary Figures S6B–E). In conclusion, altogether, these results show that, in the context of neurodegeneration, astrocytes respond to exogenously delivered NGF, both in terms of acute Ca^2+^ activity and of their asthenic neurotoxic phenotype. This contrasts with the observed deafness of astrocytes to added NGF in physiological conditions and provides a strong additional mechanistic rationale to the use of NGF agonists for treating different neurodegenerative conditions.

## 4 Discussion

Astrocytes are unique glial cells designated for the maintenance of brain homeostasis, effectively contributing to neuronal function and computation (Santello et al., 2019; Kofuji and Araque, 2021). Their critical role in neuronal function makes the discovery of novel pathways regulating their function of particular interest. In this work, we describe how NGF levels can deeply affect astrocyte morphology, transcriptome, and, ultimately, their function. The observation started from the morphological analysis of astrocytes in two mouse models of neurodegeneration, based on NGF/proNGF disequilibrium, the transgenic lines AD11 (Capsoni et al., 2000; Ruberti et al., 2000) and TgproNGF#72 (Tiveron et al., 2013). In the past, the study of these models showed that interfering with the NGF/proNGF balance can lead to neurodegeneration, suggesting a vicious cycle linking NGF dysmetabolism and Alzheimer’s disease-like neurodegeneration (Capsoni and Cattaneo, 2006; Florencia Iulita and Claudio Cuello, 2015; Cuello et al., 2019). We show here that in AD11 and TgproNGF#72 mice, astrocytes acquire an atrophic phenotype characterized by reduction in volume and branching points. This astrocytic atrophy is also found in other mouse models of neurodegeneration, such as the 3xTG and PDAPP-J20 (Beauquis et al., 2014; Polis et al., 2018), and it is thought to underlie reduced homeostatic support and neuroprotection by astrocytes, accounting for synaptic impairment and neuronal death resulting in neurodegeneration (Verkhratsky et al., 2019). Of note, we report that these morphological changes in astrocytes are well-correlated with the amount of free NGF in AD11 mice, possibly suggesting a cell-autonomous effect. By isolating astrocytes in culture conditions, we discover that the same morphological alterations found *in vivo* can, in fact, be reproduced *in vitro* when chronically depriving astrocytes of NGF *via* incubation with the antiNGF antibody mAb αD11. This antibody binds mature NGF with picomolar affinity, inhibiting its binding to both p75^NTR^ and TrkA receptors and, hence, its biological activities. Of note, the affinity of mAb αD11 for unprocessed proNGF (that shares the same binding epitope as mature NGF) is 2000-fold lower (Covaceuszach et al., 2008). Thus, in a biological mixture of mature NGF and proNGF, sub-saturating concentrations of this antibody will bind different proportions of the mature NGF and the proNGF, with a preference for binding first to mature NGF, effectively depriving astrocytes of NGF. The clear dose dependence of the effect and the maximum effect at the higher dose (which is in excess over the determined concentration of NGF in our culture conditions) indicates that proNGF is most likely the form of NGF whose blocking determines the observed effect (possibly in conjunction with mature NGF). It remains to be ascertained *via* which receptor on astrocytes the proNGF (and possibly traces of mature NGF that are still present in the mixture) is acting. Though the atrophic phenotype observed in the p75^NTR−/−^ mice (Supplementary Figure S1) suggests the prominent role of p75 for astrocytes, future work will have to address this specific point by using astrocyte-specific manipulations of NGF signaling. Our data on p75^NTR−/−^ mice have, in fact, the limitation that, in this mouse model, the observed atrophy is not necessarily cell-autonomous and might also be explained by a secondary astrocytic response to the lack of p75^NTR^ signaling in other p75^NTR^-positive cells in the brain. Moreover, the absence of atrophy in the TrkA^+/−^ is by no means definitive on the absence of a possible role for TrkA in astrocyte physiology since 1) the analysis was performed on heterozygous mice (full TrkA KO mice show early postnatal lethality and cannot be analyzed at the relevant age), allowing for a possible compensation by the other TrkA allele, and 2) a proper analysis would still require a cell-specific KO.

We then analyzed the consequences of reduced levels of NGF on astrocyte physiological functions. We focused on calcium oscillations since, in physiological conditions, they are fundamental for the release of gliotransmitters, affecting synaptic transmission, long-term potentiation, and, ultimately, behavior (Santello et al., 2019). We report that NGF deprivation *via* αD11 leads to an increase in astrocyte calcium activity that is dependent on intracellular Ca^2+^ stores. Conversely, NGF administration did not affect astrocyte calcium activity.

Then, we assessed whether depriving astrocytes of NGF causes alterations in gene expression. Transcriptomic changes induced by αD11 administration reveal an upregulation in the expression of genes associated with neuroinflammatory responses, while downregulated genes are enriched in GO terms related to DNA damage and cell division. Of interest, the top upregulated gene is cystatin F (Cst7), which is upregulated in astrocytes in the cortex of 15–18-month-old APP/PS1 mice (Orre et al., 2014). The list of 50 top genes in the previously mentioned paper shows seven additional genes in common with αD11 upregulated transcripts, i.e., Clec7a, Trem2, Ctss, Ly86, Cd52, Ccl5, and Ifi27l2a. As for the αD11 downregulated transcripts, we find two common hits, Hes5 and Ppp1r3g. Overall, this indicates that the transcriptome profile driven by αD11 resembles that present in neurodegenerative conditions. In our dataset of upregulated transcripts, we also find complement proteins (i.e., C3 and C1s2) which are known to contribute to the synaptopathy present in Alzheimer’s disease (Hong et al., 2016). Among those that are downregulated, many genes are involved in the remodeling of the extracellular matrix (ECM) (e.g., Mmp12 and Col6a3). This is of particular interest as astrocyte-derived ECM-modifying enzymes are known to contribute to plasticity in the brain (Ribot et al., 2021). Then, we performed a GSEA to compare our dataset to that published by Liddelow et al. (2017) to discover that αD11-treated astrocytes are enriched for genes associated with an A1-neurotoxic phenotype.

Prompted by this finding, we performed co-cultures of astrocytes and neurons and assessed the overall toxicity by looking at neuronal health. First, NGF deprivation exerts no effect on hippocampal neurons alone, but when combined with astrocytes, reduces NGF levels *via* αD11 and brings about neuronal death. This confirms that antiNGF-treated astrocytes acquire a neurotoxic phenotype. This toxic effect can be partially counteracted by concomitant administration of a well-established antibody fragment against oligomeric forms of Aβ peptide (Meli et al., 2009; Meli et al., 2014), scFv A13, or by diminishing glutamatergic transmission *via* KynA, identifying two potential mechanisms involved in αD11-induced neurotoxicity.

The *in vitro* data suggest that this induction of neurotoxic mechanism has a cell-autonomous component, but *in vivo*, there may be additional cellular components (neuronal or microglial) contributing to the transformation of NGF-deprived astrocytes into neurotoxic effector cells. We then conducted *in vivo* studies to understand whether NGF deprivation could also affect astrocyte physiology in the context of the brain’s circuitry. Consistent with our *in vitro* work, we uncovered that the administration of αD11 or a TrkA-based NGF scavenger, but not that of mNGF, could substantially increase calcium activity in astrocytes *in vivo*. Our *in vivo* data have the inherent limitation that they cannot prove whether these increases in calcium activity are cell-autonomous as they could be an indirect response to the action of NGF deprivation on other NGF-responsive cell types in the brain. Future work using astrocyte-specific manipulation of NGF signaling *in vivo* will have to determine whether this phenomenon is cell-autonomous in an *in vivo* condition, as we have demonstrated *in vitro*. Moreover, it would be of particular interest to understand the role of NGF signaling in astrocytes in physiological conditions and how that impinges on neuronal function. In pathological conditions, astrocyte calcium dysregulation occurs. In particular, changes in calcium oscillations are an early symptom of Alzheimer’s disease (AD) (Carter et al., 2019). Reports show that Aβ oligomers can drive Ca^2+^ waves in cultured astrocytes (Alberdi et al., 2013). A similar phenotype is seen *in vivo*, where astrocyte calcium transients are increased in synchrony and intensity in reactive astrocytes in mouse models of AD (Kuchibhotla et al., 2009; Delekate et al., 2014; Lines et al., 2022). This is of particular relevance for our study since changes in the ratio of proNGF/NGF can be found not only in AD11 mice but also in Alzheimer’s disease and in Down’s syndrome patients who develop AD with aging (Capsoni and Cattaneo, 2006; Florencia Iulita and Claudio Cuello, 2015; Cuello et al., 2019).

So, we wondered whether symmetrically, in a condition where astrocytes are reactive, administering mNGF could reverse the increase in calcium. To do so, we recorded calcium activity in astrocytes in a mouse model of AD, the 5xFAD, at a symptomatic age: in this case, remarkably, astrocytes responded to mNGF by decreasing their otherwise excessive calcium activity.

Altogether, these results show that the responses of astrocytes to the modulation of NGF signaling are asymmetric: astrocytes usually only respond to negative modulations of NGF signaling, with the notable exception of 5xFAD mice. This might be due to the fact that in physiological conditions, the set point of the NGF-to-astrocyte signaling system is homeostatically saturated, highlighting a ceiling effect, while in neurodegeneration conditions, astrocytes may become more sensitive and respond to NGF, if exposed to it.

On a similar note, we show that intranasal treatment with NGF in a mouse model of AD, the 3xTG, which is known to present astrocyte atrophy at the same early age in which the NGF treatment was performed (Yeh et al., 2011), could rescue astrocyte morphology (Supplementary Figure S6). Possible limitations of this particular experiment are 1) that the effects of an NGF treatment on a WT cohort were not assessed in this study, and 2) the lack of astrocyte-specific manipulations that would ensure a direct action of NGF on glial cells*.*

In any event, the new mechanism described here provides a novel strong rationale to develop therapeutic approaches aimed at increasing the activity of NGF in the brain, as a broad neuroprotective strategy, in the context of different neurodegenerative diseases.

**FIGURE 9:**
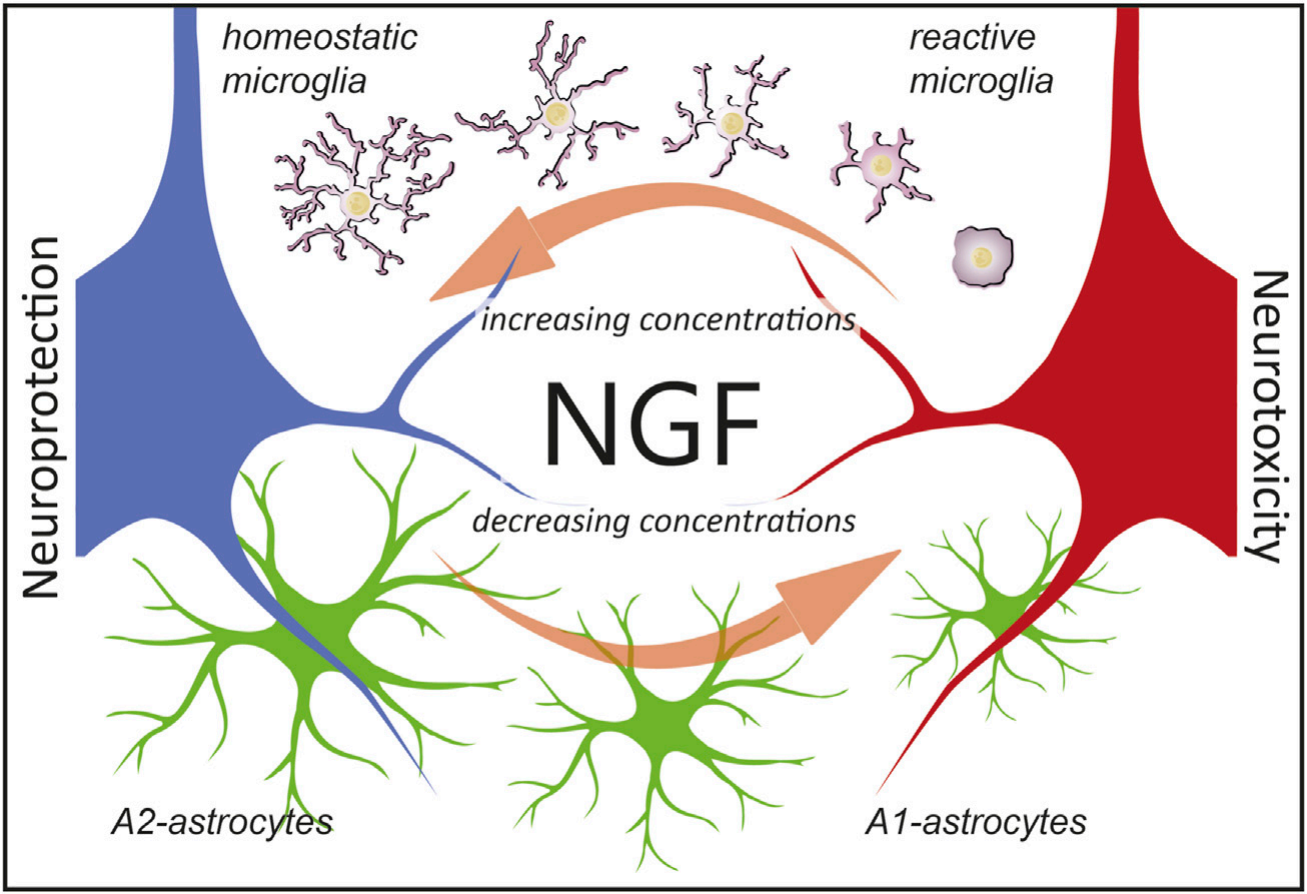
Hypothesis on the consequences of NGF signaling in glial cells. We propose that astrocytes are ambient NGF sensors. NGF levels (which include either mNGF, proNGF, or different ratios thereof) affect glial physiology. In particular, as reported in this work, decreasing concentration of NGF leads to an A1-activation of astrocytes and neurotoxicity (red neuron). On the contrary, as previously reported (Rizzi et al., 2018), increasing the concentrations of NGF steers microglia toward an anti-inflammatory phenotype, leading to neuroprotection (blue neuron). Of note, defining microglial cells as reactive or homeostatic is an oversimplification (Paolicelli et al., 2022) and we use such terms merely to indicate the neuroprotective effects of NGF *via* microglia.

One underlying complexity in the mechanistic interpretation of these experiments is that NGF exists in two different forms, mNGF and proNGF, which coexist in the brain *in vivo* (Fahnestock et al., 2001) and whose relative levels need to be finely regulated (Capsoni and Cattaneo, 2006; Florencia Iulita and Claudio Cuello, 2015; Cuello et al., 2019). It has been established, under controlled *in vitro* conditions, that the combination of proNGF and NGF acts, in a distinct way from either NGF or proNGF alone, depending on the proNGF/NGF ratio, emphasizing the fact that when both NGF and proNGF downstream pathways are activated, conflict, synergism, and/or cancellation may occur (Arisi et al., 2014). This most likely involves interactions between the two signaling pathways, both at the receptor level, to their specific downstream functional consequences. It will be of particular interest, in future work, to investigate the effect of proNGF alone, or of NGF/proNGF mixes in astrocytes, to dissect the signaling whereby these cells sense, and functionally react to, the ambient NGF/proNGF levels. Another limitation that future work will address is to add microglia cells in the picture of the *in vivo* phenomena described here. It is, in fact, well-known that astrocytes are very much influenced by microglial cells (Liddelow et al., 2017). In turn, previous work has demonstrated that NGF can act on microglial cells by steering them toward a neuroprotective phenotype (Capsoni et al., 2017; Rizzi et al., 2018). As such, NGF could orchestrate the response of both microglia and astrocytes to either injury or neurodegeneration (Figure 9). This further points to the relevance of NGF-based therapies for neurodegenerative disorders (Cattaneo and Capsoni, 2019) in which both astrocytes and microglia are involved (Verkhratsky et al., 2010; Ardura-Fabregat et al., 2017; Monterey et al., 2021).

## Data Availability

The datasets presented in this study can be found in online repositories. The names of the repository/repositories and accession number(s) can be found at: https://www.ncbi.nlm.nih.gov/geo/, GSE108103.
