# Supplementary material for: Reduced levels of NGF shift astrocytes toward a neurotoxic phenotype
Source: Front Cell Dev Biol. 2023 Apr 18;11:1165125. doi: 10.3389/fcell.2023.1165125 (PMC10151754; doi:10.3389/fcell.2023.1165125)
Supplement: Supplementary file 2 [file Image1.PDF]

Supplementary Material

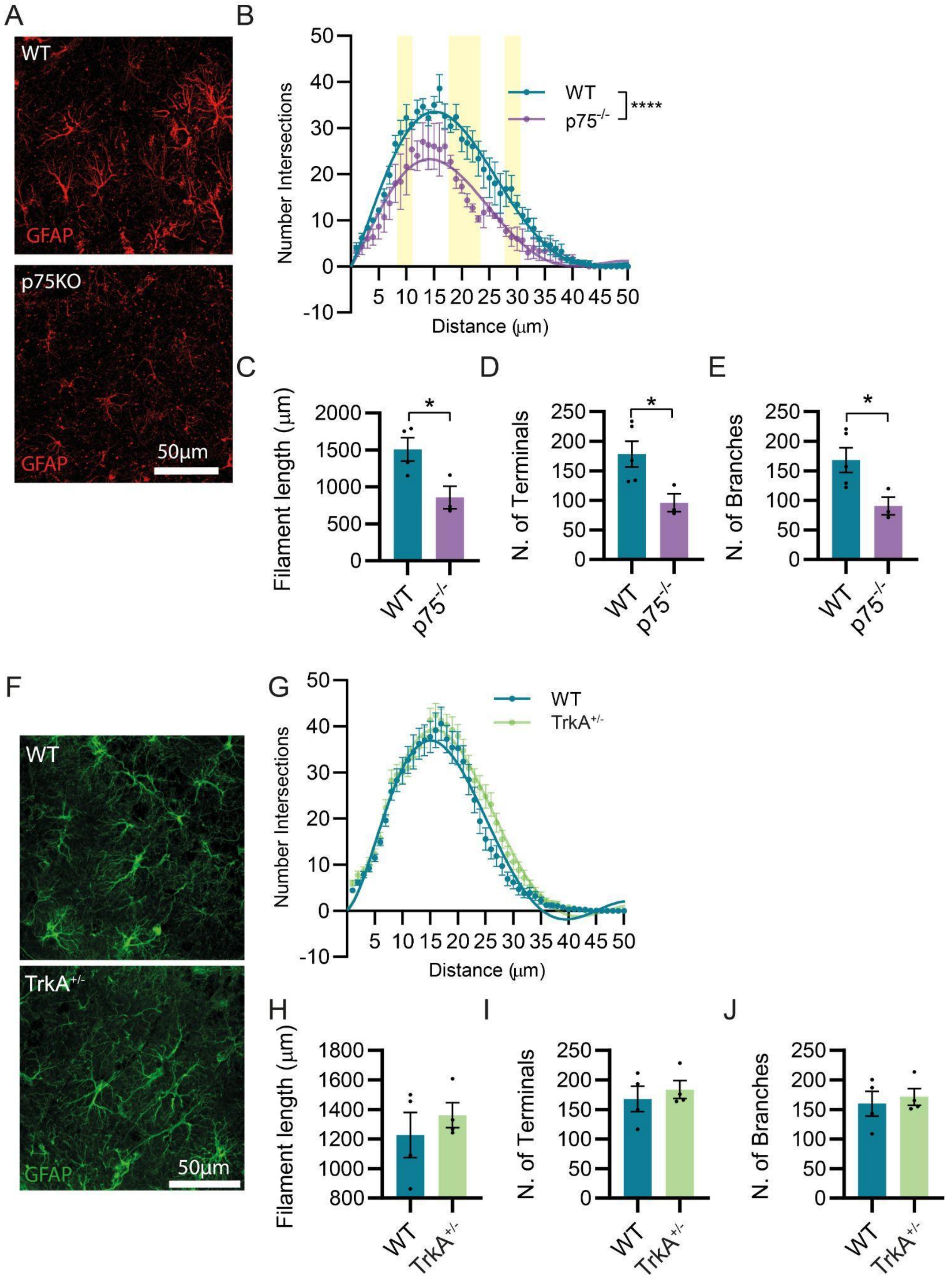

Supplementary Material

Supplementary Figure 1. Alteration in astrocyte morphology in p75$^{-/-}$ mice but not in TrkA$^{+/-}$ mice. (A) Representative images of GFAP-labelled astrocytes in 2 months old p75$^{-/-}$ and control mice. (B) Sholl analysis of astrocyte morphology in p75$^{-/-}$ and control mice (two-way ANOVA; $p<0.0001$, n=3 animals per group, the yellow box indicates significance in Sidak's multiple comparisons test). (C-E) Barplots representing the total filament length, the number of terminals, and the number of branching points in p75$^{-/-}$ and control mice, respectively (n=3 animals per group). (C) (Two-tailed Unpaired t-test; p=0.0347). (D) (Two-tailed Unpaired t-test; p=0.0381). (E) (Two-tailed Unpaired t-test; p=0.0398). (F) Representative images of GFAP-labelled astrocytes in 6 months old TrkA$^{+/-}$ and control mice. (G) Sholl analysis of astrocyte morphology in TrkA$^{+/-}$ and control mice (two-way ANOVA). (H-J) Barplots representing the total filament length, the number of terminals, and the number of branching points in TrkA$^{+/-}$ and control mice, respectively (Two-tailed Unpaired t-test; n=3 animals per group).

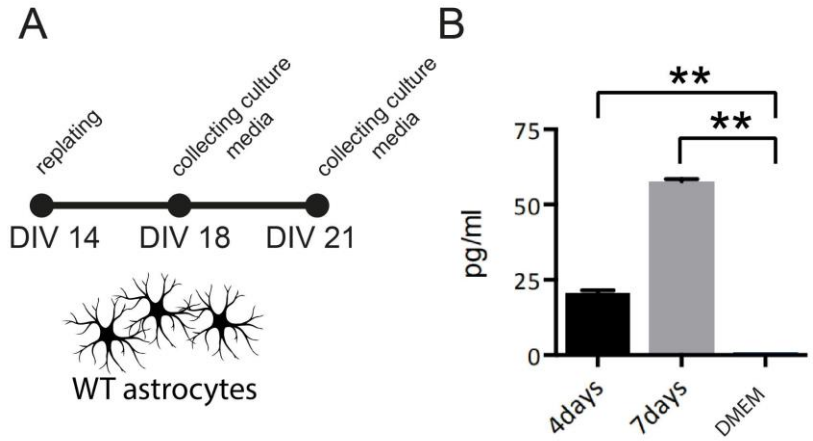

Supplementary Figure 2. Astrocytes secrete (pro) NGF *in vitro*. (A) Timeline of the collection of the media from astrocyte cultures for ELISA analysis (B) (D) Quantification of the amount of NGF in astrocytic culture medium (one-way ANOVA, medium vs 4 days, $p<0.001$, medium vs 7 days, $p<0.001$, n=3 independent experiments).

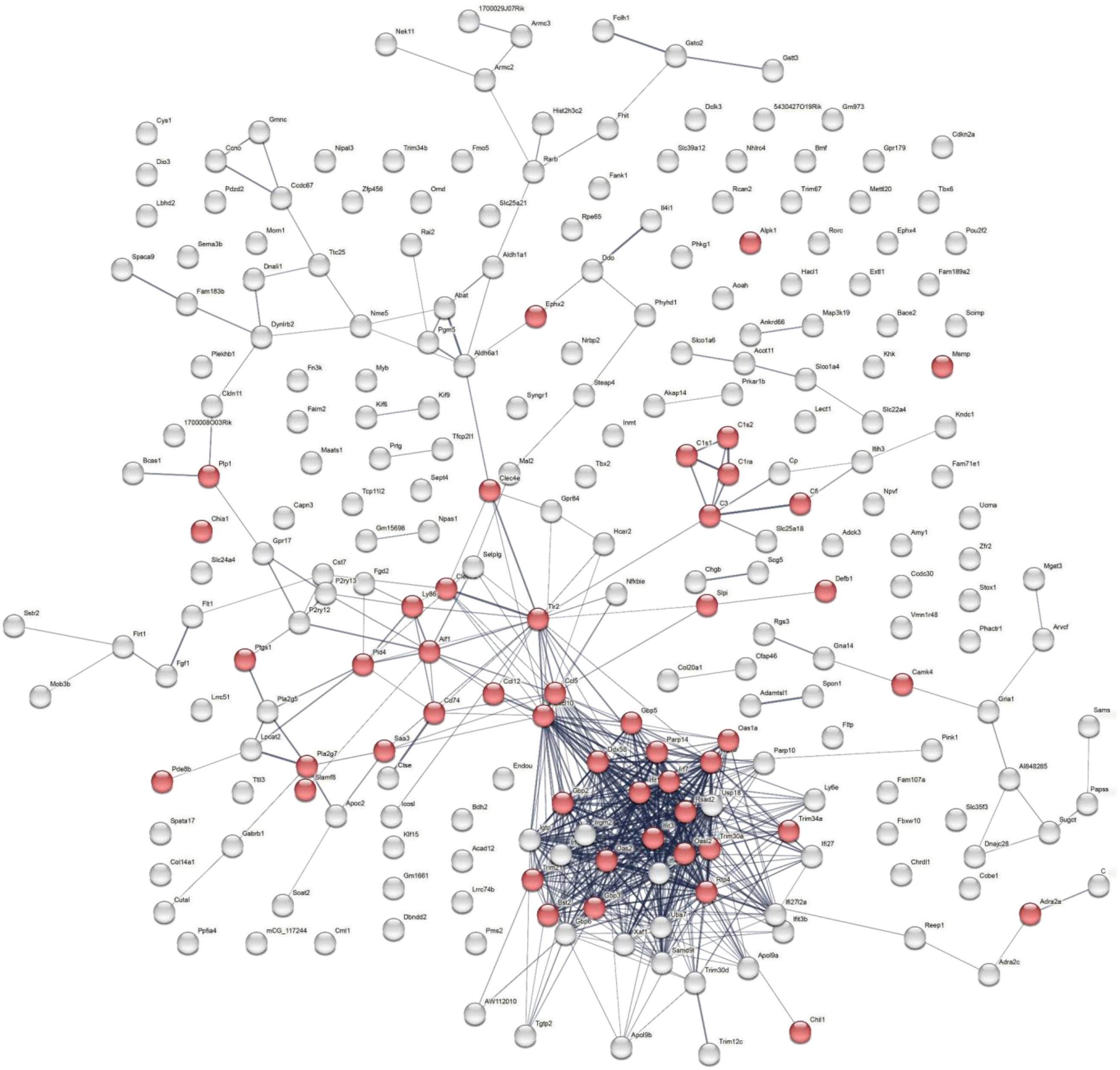

Supplementary Figure 3. StringDB analysis of transcriptomic data for UPREGULATED genes. The network was obtained by StringDB using the top 300 most upregulated genes from the intersection gene list (n=1129). Immune system-related genes are in red.

Supplementary Material

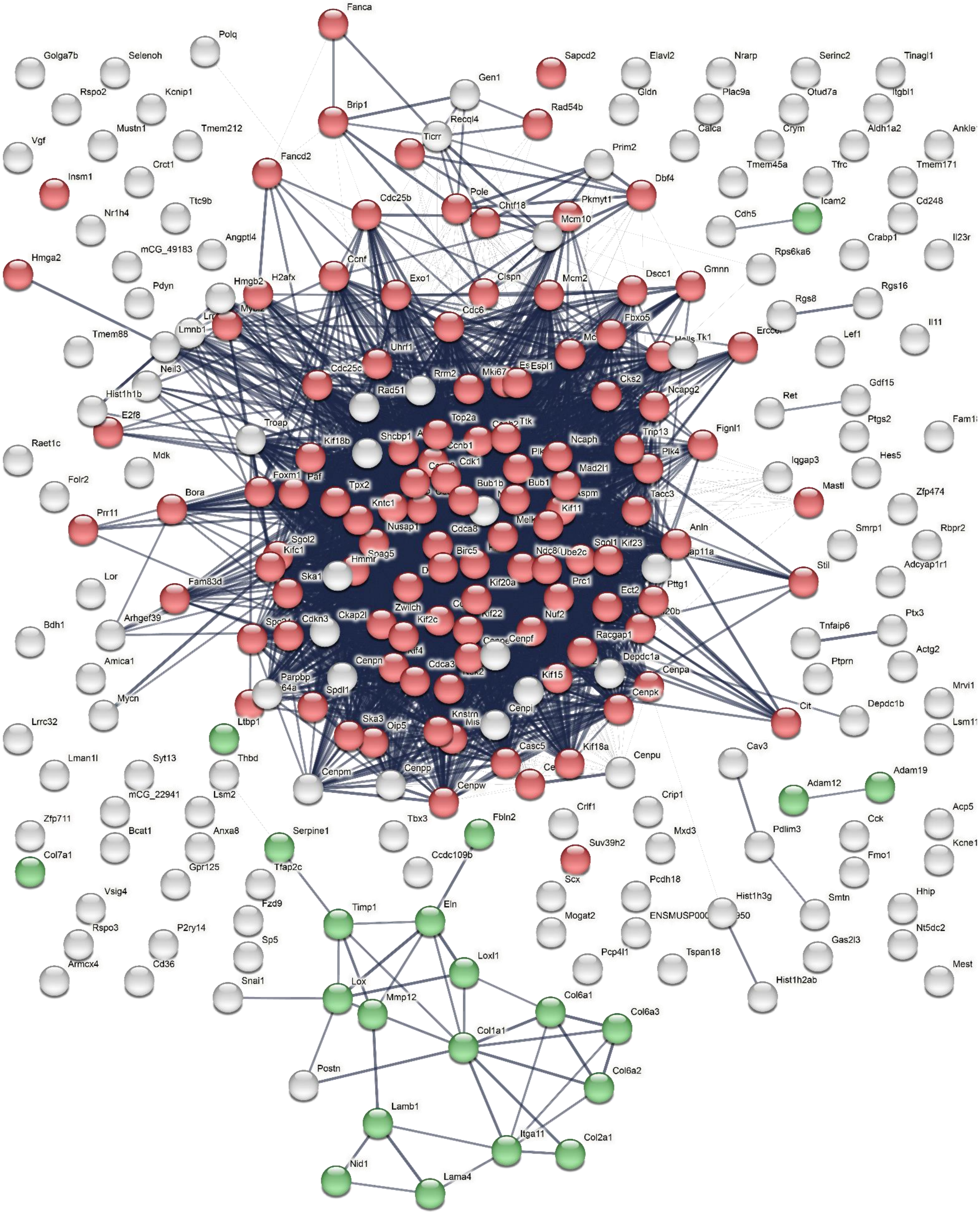

Supplementary Figure 4. StringDB analysis of transcriptomic data for downregulated genes. The network was obtained by StringDB using the top 300 most downregulated genes from the intersection gene list (n=1128). Cell cycle-related genes are in red, while Extracellular matrix-related genes are in green.

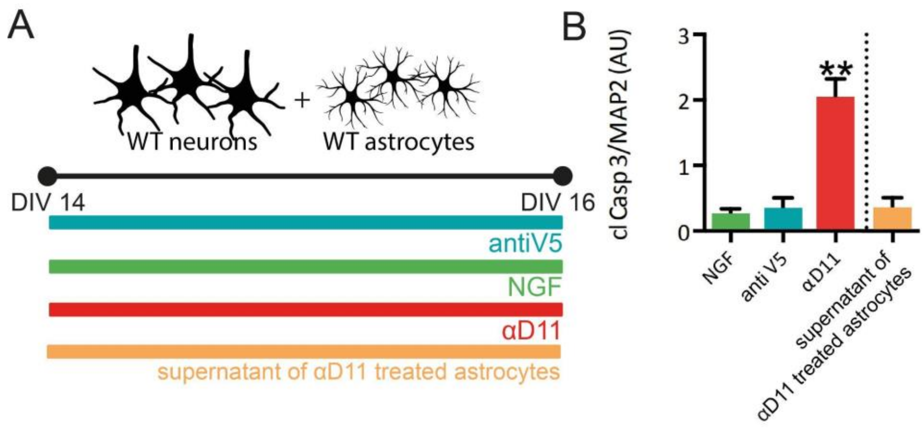

Supplementary Figure 5. Cleaved Caspase-3 immunostaining in NGF-deprived astrocyte-neuron co-culture. (**A**) Timeline of the experiment. Astrocyte-neuron co-culture were treated for 48 hours with a control antibody (antiV5), αD11, NGF or the supernatant of αD11-treated astrocytes (**B**) Quantification of the mean fluorescence of Cleaved Caspase-3 immunostaining in MAP2 stained neurons (one-way ANOVA, V5 vs αD11: $p<0.001$).

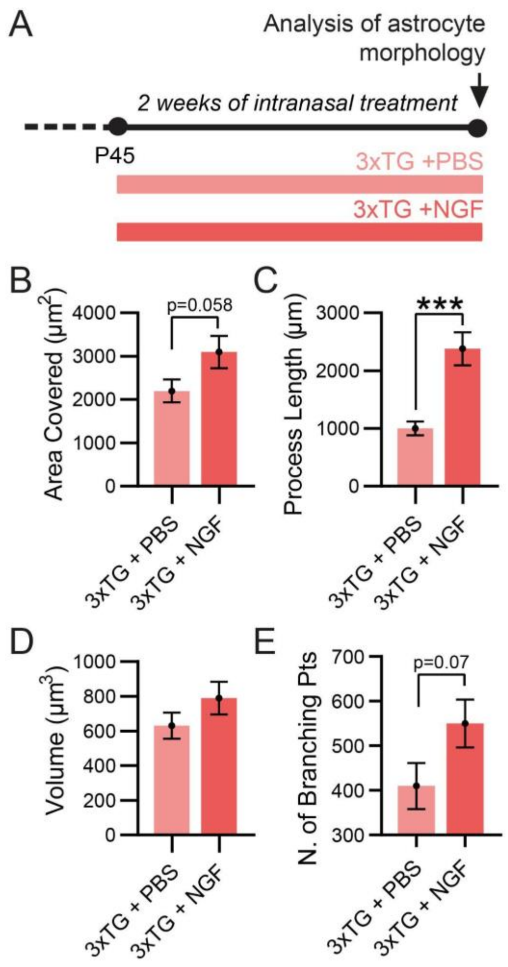

Supplementary Figure 6. Rescue of astrocyte asthenic morphology in 3xTG mice via intranasal NGF administration. (**A**) Timeline of the experiments. Animals were treated with either NGF or PBS times per week for two weeks from P45 to P60, then sacrificed to analyze astrocyte morphology. (**B**) Area covered by the astrocyte processes (Two-tailed Unpaired t-test; p=0.0584). (**C**) Total process length (Two-tailed Unpaired t-test; p=0.0001). (**D**) Total volume of astrocyte processes (Two-tailed Unpaired t-test; p=0.1977). (**E**) Number of branching points (Two-tailed Unpaired t-test; p=0.0713).
